# Assessing the causes and consequences of gut mycobiome variation in a wild population of the Seychelles warbler

**DOI:** 10.1186/s40168-022-01432-7

**Published:** 2022-12-28

**Authors:** Sarah F. Worsley, Charli S. Davies, Maria-Elena Mannarelli, Jan Komdeur, Hannah L. Dugdale, David S. Richardson

**Affiliations:** 1 School of Biological Sciences, University of East Anglia, Norwich Research Park, Norfolk, NR4 7TJ UK; 2 NERC Biomolecular Analysis Facility, Department of Animal and Plant Sciences, University of Sheffield, Sheffield, S10 2TN UK; 3 Groningen Institute for Evolutionary Life Sciences (GELIFES), University of Groningen, P.O. Box 11103, 9700 CC Groningen, The Netherlands; 4 Faculty of Biological Sciences, School of Biology, University of Leeds, Leeds, LS2 9JT UK; 5 Nature Seychelles, Roche Caiman, Mahé, Republic of Seychelles

**Keywords:** Gut microbiome, Fungi, Major histocompatibility complex, Genetic variation, Fitness, *Acrocephalus sechellensis*

## Abstract

**Background:**

Considerable research has focussed on the importance of bacterial communities within the vertebrate gut microbiome (GM). However, studies investigating the significance of other microbial kingdoms, such as fungi, are notably lacking, despite their potential to influence host processes. Here, we characterise the fungal GM of individuals living in a natural population of Seychelles warblers (*Acrocephalus sechellensis*). We evaluate the extent to which fungal GM structure is shaped by environment and host factors, including genome-wide heterozygosity and variation at key immune genes (major histocompatibility complex (MHC) and Toll-like receptor (TLR)). Importantly, we also explore the relationship between fungal GM differences and subsequent host survival. To our knowledge, this is the first time that the genetic drivers and fitness consequences of fungal GM variation have been characterised for a wild vertebrate population.

**Results:**

Environmental factors, including season and territory quality, explain the largest proportion of variance in the fungal GM. In contrast, neither host age, sex, genome-wide heterozygosity, nor *TLR3* genotype was associated with fungal GM differences in Seychelles warblers. However, the presence of four MHC-I alleles and one MHC-II allele was associated with changes in fungal GM alpha diversity. Changes in fungal richness ranged from between 1 and 10 sequencing variants lost or gained; in some cases, this accounted for 20% of the fungal variants carried by an individual. In addition to this, overall MHC-I allelic diversity was associated with small, but potentially important, changes in fungal GM composition. This is evidenced by the fact that fungal GM composition differed between individuals that survived or died within 7 months of being sampled.

**Conclusions:**

Our results suggest that environmental factors play a primary role in shaping the fungal GM, but that components of the host immune system—specifically the MHC—may also contribute to the variation in fungal communities across individuals within wild populations. Furthermore, variation in the fungal GM can be associated with differential survival in the wild. Further work is needed to establish the causality of such relationships and, thus, the extent to which components of the GM may impact host evolution.

Video Abstract

**Supplementary Information:**

The online version contains supplementary material available at 10.1186/s40168-022-01432-7.

## Background

The vertebrate gut microbiome (GM) is a diverse microbial ecosystem of fundamental importance to the host. Species within the GM make critical contributions to many aspects of host biology, from nutrient acquisition to influencing host behaviour, immunity, and development [1–3]. GM structure can vary substantially amongst individuals living within the same natural population [4–6]. Given the importance of microbial species to host function, such variation may have significant consequences for traits associated with host fitness, including disease susceptibility [7, 8], survival [9, 10], and reproductive success [11, 12]. However, most GM research is biassed towards studying bacteria, the dominant taxonomic group (according to the proportion of sequencing reads) in the vertebrate gut [13]. In contrast, few studies have investigated the extent to which other microbial kingdoms in the GM, such as viruses, archaea, and microbial eukaryotes, vary across individuals living in wild vertebrate populations, and the potential drivers of that variation [14–16]. Furthermore, none of these studies has investigated the fitness consequences associated with variation in non-bacterial GM communities.

Fungi are an important but understudied component of the vertebrate GM, being present at lower abundance than bacterial taxa [13]. Despite this, evidence is accumulating that host-associated fungal communities, known as “the mycobiome”, can impact host health by contributing to important host processes. In the gut, fungal species can play an important role in the digestion of complex dietary components [17, 18], as well as the development and activity of the host immune system [19–21]. For example, laboratory experiments have demonstrated that increased fungal diversity in the GM can reduce the severity of diseases such as colitis in captive mice (*Mus musculus*) [22, 23], and certain fungal species can functionally replace intestinal bacteria in mitigating mucosal tissue injury [19].

Although numerous studies on captive animals have indicated the potential importance of the gut mycobiome to host health, very few studies have investigated gut mycobiome variation across individuals living in wild populations and the possible drivers of this variation [14, 24, 25]. Indeed, although a handful of studies have shown that environmental factors, such as season and habitat differences, influence fungal GM composition [14, 24, 25], there are no studies investigating the extent to which host genetic differences are associated with fungal GM variation across individuals living in wild populations. Moreover, we are not aware of any studies that have looked at the potential consequences of this mycobiome variation for host fitness components (e.g. differential survival) in the wild. This is despite growing evidence that captivity can significantly alter, and in many cases simplify, the GM [26, 27], including the mycobiome [21, 28]. Wild animals are exposed to highly complex and dynamic environments which are very difficult to replicate in captivity, but which shape the GM and interact with host fitness [29]. Furthermore, inbred captive hosts harbour reduced levels of genetic variation and, thus, are not fully representative of wild populations [29, 30]. As such, studying the extent to which host-associated mycobiome communities vary under natural conditions, the possible drivers of this variation, and the consequences for host fitness will be crucial if we are to fully understand the dynamics of the GM and the functional significance of the mycobiome to the host.

Variation in immune receptor genes, which recognise specific microbial antigens and activate an immune response, may play a key role in driving inter-individual differences in GM structure. A number of innate immune receptors respond to commensal and pathogenic fungi at mucosal surfaces, including C-type lectins, interleukins, and Toll-like receptors [19, 31]. Similarly, components of the adaptive immune system also interact with fungi and may play a key role in sculpting the microbiome. One such component is the major histocompatibility complex (MHC), a gene family present in virtually all vertebrate species [32–34]. MHC genes encode cell-surface glycoproteins which bind to protein antigens, including those derived from intestinal microbes. The resulting MHC-peptide complex is then presented to T-lymphocytes which, depending on the nature of the antigen, trigger an appropriate immune or tolerogenic response [35–37]. There are two major classes of MHC genes which process antigens via distinct pathways [33, 38]. MHC class I (MHC-I) genes are expressed on almost all somatic cells and predominantly process intracellular peptides (e.g. those derived from viruses). In contrast, MHC class II (MHC-II) genes are expressed on the surface of antigen-presenting cells and primarily bind to extracellular peptides, such as those derived from bacterial and fungal cells in the intestinal tract.

MHC genes exhibit extreme polymorphism, with many allelic variants existing in natural populations [32, 34, 39]. These polymorphisms can determine the range of antigen peptides (and therefore microbial species) that are recognised by the immune system [32]. As such, several studies have demonstrated that variation in MHC genotype may drive inter-individual differences in the bacterial GM [4, 40, 41]. For example, a previous study on the Seychelles warbler (*Acrocephalus sechellensis*) [4] demonstrated that specific MHC-I and MHC-II alleles are associated with shifts in the diversity and composition of the bacterial GM. In contrast, very little is known about the extent to which commensal fungal communities are shaped by variation at the MHC. Most research has focussed on specific fungal pathogen species, such as susceptibility to *Batrachochytrium dendrobatidis* infection in amphibians [42–44]. Comparatively few studies have looked at the influence of MHC variation on wider host-associated fungal communities [45, 46], and as far as we are aware, there are no studies investigating the relationship between MHC variation and fungi in the GM.

In this study, we investigate the environmental and genetic drivers of fungal GM variation in a natural population of the Seychelles warbler. We also investigate the relationship between fungal GM differences and host survival; to our knowledge, this is the first time this has been characterised in a natural vertebrate species. Importantly, the Seychelles warbler population on Cousin Island is closed, with virtually no inter-island dispersal, thus enabling accurate measures of survival to be achieved [47]. Although the population harbours reduced genetic variation as a result of a population bottleneck [48], variation still exists at the MHC-I and MHC-II loci [4, 49] and has been linked to survival differences [50]. In the Seychelles warbler, inter-individual variation in bacterial GM structure has been associated with age, sex, sampling season, genome-wide heterozygosity, and, importantly, MHC genotype [4]. Furthermore, differences in bacterial GM composition have been associated with survival [9]. Here, we expand our investigation of the GM to the mycobiome, by sequencing the fungal internal transcribed spacer subregion two (ITS2) ribosomal DNA.

We first test whether differences in fungal GM structure are associated with environmental variables, including season, territory quality, and time of day. We also test for an association with host variables, including sex, age, and genome-wide heterozygosity. Following this, we restrict our analysis to those individuals with complete immunogenetic data to test whether MHC diversity and/or specific MHC alleles are associated with variation in the diversity or composition of the fungal GM. In addition, we test for an association between mycobiome structure and *TLR3* genotype, one of the few TLR loci for which functional variation has been maintained in the Seychelles warbler [51, 52]. The *TLR3* genotype is correlated with host survival and reproductive success in this population [51]. Thus, although the *TLR3* gene is thought to play a role in viral sensing [53], it may have an indirect impact on fungal communities, by influencing host susceptibility to viral infection or other aspects of host health. Finally, using the full dataset, we test for an association between variation in fungal GM structure and the probability of host survival to the next breeding season.

## Methods

### Study species and sample collection

The study was carried out on the population of Seychelles warblers inhabiting Cousin Island (29 ha; 04° 20′ S, 55° 40′ E), which has been monitored as part of a long-term research project since 1985 [51, 54, 55]. The population on Cousin consists of ca. 320 adult individuals which are distributed across ca. 115 year-round territories [55, 56]. Almost all individuals (> 96%) have been individually marked with a unique combination of a British Trust for Ornithology (BTO) metal ring and three plastic colour rings [57]. A population census is carried out twice a year in the minor (January to March) and major (June to September) breeding seasons [58]. As the annual resighting probability of adult individuals is high (98% ± 1% SE) [50] and inter-island dispersal is virtually absent [47], individuals not seen during a breeding season can be confidently assumed dead. Seychelles warblers have a median life expectancy at fledging of 5.5 years and a maximum recorded lifespan of 19 years [55, 59]. Since they lack natural predators and experience limited human disturbance, extrinsic mortality is lower than in many passerine species living in temperate regions [60]. Indeed, the average annual survival probability is exceptionally high in adults (0.84 ± 0.04 SE) and juveniles (0.61 ± 0.09) [60].

Individuals were caught in mist nests. Age was estimated based on a combination of lay, hatch, or fledge date or, if these dates were unknown, estimated from eye colour which changes from grey in fledglings to red-brown in adult individuals [54]. The mean age of individuals was 2.46 ± 2.88 SD (range 0.12–15.78 years). Blood samples were taken via brachial venipuncture and stored in absolute ethanol at 4 °C for later molecular analysis. As Seychelles warblers are insectivorous, an index of territory quality was calculated based on the number of insect prey available, the territory size, and foliage cover in an individual’s territory during that breeding season [54]. For territories with missing quality measures in a season, quality was calculated as the mean from the preceding and following breeding periods [as in 60].

Faecal samples were collected as described previously [see 4]. Briefly, captured birds were placed into a disposable, flat-bottomed paper bag containing a sterilised weigh boat protected by a metal grate; this set-up allows faecal matter to fall into the tray whilst minimising the possibility of contamination from the bird’s surface. Each bird was removed from the bag after defecation (or 30 min in the case of no defecation). The faeces were then collected into a sterile microcentrifuge tube containing 1 ml of absolute ethanol. Control samples were collected from empty collection bags and the fieldworker’s hands to capture possible sources of contamination (*n* = 7). All samples were stored at 4 °C for the remainder of the field season, before transferring to − 80 °C for long-term storage. The time kept at 4 °C was controlled for in all downstream analyses. A total of 277 faecal samples (one per individual) were taken across five sampling seasons (the minor breeding seasons of 2018 and 2019 and the major breeding seasons of 2017, 2018, and 2019) spanning 3 years. The survival of sampled individuals was assessed during the following breeding season (i.e. up to the minor breeding season of 2020).

### Molecular methods

DNA was extracted from blood samples using the DNeasy Blood and Tissue kit (Qiagen, Crawley, UK) according to the manufacturer’s protocol. Molecular sexing was carried out using an established PCR-based method [59, 61]. Individuals were genotyped at 30 polymorphic microsatellite loci [57, 59], and standardised individual microsatellite heterozygosity (*H*_*s*_) was calculated using *genhet* 3.1 [62] in R 4.1.1 [63]. Variation at a single non-synonymous SNP within the leucine-rich repeat domain of *TLR3* exon 4, determined as part of a separate study, resulted in three genotypes (*TLR3*^*AA*^, *TLR3*^*AC*^, or *TLR3*^*CC*^) (see [51] for details). MHC-I exon 3 and MHC-II exon 2 were amplified and sequenced using Illumina MiSeq technology as part of a previous study (see [4] for methods). Complete MHC genotype data were available for 202 out of the 277 individuals with GM samples in the present study. Here, the term “allele” refers to different variants amplified for each class of MHC. Genotyping identified ten MHC-1 alleles that were present in > 5% but < 95% of individuals, and a further 10 alleles had a frequency of < 5%. Based on all alleles, individuals carried a mean of five MHC-I alleles (range 2–7 alleles). Three MHC-II alleles were present in > 5% but < 95% of individuals. A further two alleles were present in almost all individuals (> 97%), and nine alleles had a frequency of < 5%. Individuals carried a mean of 2.9 MHC-II alleles (range 1–5 alleles). All alleles were included in MHC diversity calculations but not in analyses of allele presence/absence, for which only alleles present in > 5% but < 95% of individuals were included.

### Microbiome extraction and sequencing of the fungal community

Total genomic DNA was extracted from faecal samples and collection controls using the DNeasy PowerSoil Kit (Qiagen), following an optimised version of the manufacturer’s instructions (see [4] for detailed methods). As part of this protocol, samples were lysed using a combination of chemical lysis and bead beading. Chemical lysis was performed by means of the C1 lysis buffer (included in the kit). For bead beating, each sample was added to a PowerBead tube (provided) and secured to a vortex adapter; samples were then vortexed at maximum speed for 10 min. Modifications to the kit instructions included heating samples in the C1 buffer (65 °C for 10 min) prior to bead beating to improve lysis and eluting DNA in a final volume of 60 µl elution buffer. Samples were randomised across extractions. The bacterial component of the same extractions was sequenced as part of previous studies [4, 9]. Five randomly selected faecal samples were extracted twice so that the consistency of the extraction method for isolating fungal DNA could be assessed. One negative control was carried out per extraction kit using a blank sterile swab (*n* = 5 extraction blanks in total). Two types of positive control were also extracted to verify the completeness of the extraction protocol and reproducibility of sequencing methods. The first was extracted from a D6300 Microbial Community Standard (ZymoBIOMICS) which contains two fungal species (*Saccharomyces cerevisiae* and *Cryptococcus neoformans*) that are typically hard to lyse. The second was extracted from a synthetic fungal mock community containing 12 unique sequences [64]. The DNA concentration in each extraction was quantified using a Qubit dsDNA High Sensitivity Assay kit (Invitrogen).

Following DNA extraction, the ITS2 subregion of the fungal ITS rRNA was amplified and sequenced using a universal tail tag dual index barcoding approach [65]. For the first-round PCR, amplification was carried out in 96-well plates; 25-μl reactions were used for each sample. The primers gITS7_fw and ITS4_rev (Additional file 1: Table S1) were used for amplification [66, 67]. Primers were adapted to include recognition sequences at the 5′ end so that a secondary nested PCR could be carried out to incorporate Illumina adapter sequences and barcodes. The PCR mix contained 12.5 μl of 2 × Kapa HiFi Hotstart ReadyMix (Roche), 0.75 μl each of the forward and reverse primers (10 μM concentration), 8 μl of sterile water, and 3 μl of DNA template per reaction. Thermocycler conditions were as follows: 95 °C for 3 min; 20 cycles of 98 °C for 20 s, 55 °C for 15 s, and 72 °C for 60 s; and 72 °C for 10 min. One negative control (using 3 µl of sterile water instead of DNA template) was carried out per 96-well plate (*n* = 4 in total). First-round PCR products were then submitted to the Centre for Genomic Research (CGR), Liverpool, where they were cleaned using AMPure beads and entered into a second-round PCR (as described in [65]). Briefly, an Illumina index primer set (i7 and i5) comprising a unique 8 nucleotide index sequence and bases complementary to the sequence introduced in the first-round PCR was used to tag the library [65]. Amplification was carried out using the same reagents and conditions as in the first-round PCR, with 20 cycles. Amplicons were quantified via Qubit, and fragment distributions were assessed using an Agilent DNA high-sensitivity kit (2100 Bioanalyzer, Agilent). Amplicons were then pooled on an equimolar basis. The amplicon pool was then purified, and size was selected within the 300–700 bp range using PippinPrep. The fragment distribution was further checked via qPCR. Libraries underwent 2 × 300 bp paired-end sequencing on an Illumina MiSeq platform (Illumina, San Diego). Each library was run twice to increase sequencing depth; data from both runs were combined at the data pre-processing stage. A total of 300 samples were sequenced. This included 277 faecal samples and 5 extraction duplicates (thus, 282 faecal extractions in total), along with 7 collection controls, 2 positive controls (the D3600 standard and the synthetic mock community), 5 blank extraction controls, and 4 PCR negative controls.

### Bioinformatic processing of fungal reads

The raw reads were trimmed at the CGR using Cutadapt 1.2.1 [68] to remove Illumina adapter sequences. Reads were further trimmed using Sickle 1.200 [69] with a minimum window quality score of 20. Data for the same sample (across the two MiSeq runs) were concatenated at this point. Upon receipt at UEA, sequences were imported into QIIME2 2019.10 [70] for further processing. The DADA2 plugin [71] was used to trim 6 base pairs from the 5′ end of reads to remove low-quality base calls. Reads were not truncated further as the ITS2 subregion can vary substantially in length across different fungal taxa [72]. Amplicon sequencing variants (ASVs) were inferred for each sample, followed by dereplication, paired-end joining, and the removal of chimeras and singletons (ASVs represented by a single read in the entire dataset). Two faecal samples which had very low read counts prior to DADA2 filtering contained zero reads after this step and so were removed. A total of 21,868,562 reads (mean per sample of 72,895.207 ± 88,523.704 SD) remained across 298 samples. Following this, ASVs were taxonomically classified by training a naïve-Bayes classifier on the developer version of the QIIME-compatible release of the UNITE database (version 8.3) [73]. A total of two ASVs were detected in the D6300 ZymoBIOMICs positive control; these corresponded to the two constituent fungal species *Saccharomyces cerevisiae* and *Cryptococcus neoformans*. Additionally, 12 ASVs with > 50 reads were detected in the synthetic mock community; these corresponded to the 12 synthetic sequences, suggesting that the extraction and sequencing methodologies had accurately captured this community. ASV and taxonomy tables were exported and further processed using *phyloseq* 1.36.0 [74] in R 4.1.1 [63].

Before conducting downstream analysis, sequences were filtered to remove ASVs that were unassigned at the phylum level. Additionally, potential contaminants were identified and filtered using the prevalence method in *DECONTAM* 1.12.0 [75]. First, laboratory contaminants were identified using PCR and extraction blanks as a reference; 7 ASVs were removed. Several of these were species of *Candida* which are known to contaminate reagents [76]. The second filtering step aimed to identify potential contaminants introduced at the sampling stage; collection controls taken from observer hands or from sample bags were used as a reference for this step. A total of 104 ASVs were filtered as possible contaminants—many of these were at a higher prevalence in low biomass samples. Following filtering, a total of 3441 fungal ASVs were detected across 279 faecal samples (a further low biomass faecal sample had zero reads following filtering). Sample completeness and rarefaction curves were generated using the R package *iNEXT* 2.0.20, with 50 bootstrap replicates per sample [77]. Sample completeness plateaued at approximately 5000 reads (Additional file 1: Fig. S1); all faecal samples with fewer than 5000 reads were therefore excluded from downstream analyses (14 samples). As the final filtering step, ASVs with fewer than 50 reads in total across all samples were also removed prior to downstream analysis, as these may represent possible sequencing errors. A total of 2540 ASVs were retained across 265 faecal sample extractions (mean ASVs per faecal sample = 51.69 ± 27.90 SD).

### Statistical analyses

#### Alpha diversity analysis

Samples were rarefied to a depth of 5000 reads prior to calculating alpha diversity metrics to control for variation in library size across samples. A total of 2530 fungal ASVs remained across 265 samples following rarefaction. The observed ASV richness in each sample and the Shannon diversity index (which accounts for ASV abundance in a sample) were calculated using *phyloseq* 1.36.0 [74]. To assess whether the DNA extraction method was consistent, pairwise Euclidean distances were calculated based on the observed ASV richness in samples that had been extracted twice. The Kruskal–Wallis test was used to compare how pairwise distances varied according to the type of sample comparison, i.e. comparisons of duplicate extractions from the same sample, versus comparisons of extractions from different samples. Following this analysis, duplicates were filtered such that only the extraction with the highest read count was retained in downstream analyses, excepting one set of duplicates in which both were filtered to avoid pseudoreplication as this bird had been sampled again on a separate occasion (259 samples remained after filtering).

Following the calculation of alpha diversity metrics, correlations between fungal and bacterial alpha diversity metrics for the same samples were compared. We then analysed different subsets of our data using an information-theoretic (model averaging) approach [78, 79]. We first analysed the full dataset (*n* = 259 samples/individuals). Four “floater” individuals were removed prior to analysis as these had no assigned territory (*n* = 255 samples/individuals remaining). To establish whether fungal alpha diversity varied according to host or environmental factors (not including immunogenetic variables), a global linear model with a Gaussian distribution was constructed using *stats* 4.1.1 [63]. Shannon diversity was used as the response variable. Host sex, age, genome-wide heterozygosity (*H*_*s*_), breeding season (major or minor), territory quality, and the amount of time the sample was stored at 4 °C prior to freezing were included as independent variables. Squared terms were also included for age and *H*_*s*_ but were subsequently removed due to a lack of significance (*P* > 0.05) and to enable the interpretation of first-order effects. Continuous variables were centred and scaled using *arm* 1.12.2 [80]. Collinearity between independent variables was checked using the *vif* function in *car* 3.0.12 [81]; vifs were < 2 for all variables in the model. Further model diagnostics were carried out using *DHARMa* 0.4.5 [82]. The *dredge* function in *MuMIn* 1.44.3 [83] was used to build all plausible models and rank them by AICc scores. All models with AICc scores within 7 of the top model were included in the final model set and used to obtain the conditional averaged estimates [78]. To assess whether results were consistent across different alpha diversity metrics, a second model was run with observed ASV richness as the response variable; a generalised linear model (GLM) was constructed with a negative binomial distribution using glm.nb in *MASS* 7.3.54 [84] to account for ASV counts being over-dispersed. The same independent variables were included in this model.

For the second set of analyses, the dataset was restricted to only include individuals with complete MHC and *TLR3* genotype data (*n* = 189 samples/individuals). Two model types were generated for each alpha diversity metric; this was to establish whether fungal alpha diversity varied according to (1) MHC diversity and (2) the presence/absence of specific MHC alleles. The first model contained MHC-1 and MHC-II diversities as independent variables, as well as their squared terms, since optimal diversity could be more beneficial [85]. The squared terms were subsequently removed due to a lack of significance (*P* > 0.05) and to enable the interpretation of first-order effects. The second model included the presence/absence of each MHC allele. For MHC-I, these were *Ase-ua1*, *3–8*, and *11*. *Ase-ua10*, and *Ase-ua1* were highly correlated (98% co-occurrence), so only *Ase-ua1* was included in the analysis. The MHC-II alleles were *Ase-dab3*, *4*, and *5*. Both model types also included heterozygosity (*H*_*s*_) and *TLR3* genotype (*TLR3*^*AA*^, *TLR3*^*AC*^, or *TLR3*^*CC*^). Host sex, age, breeding season (major or minor), territory quality, time of day (AM or PM), and time stored at 4 °C were also controlled for in each model. Since squared terms for age and *H*_*s*_ were not significant predictors in the larger model set, these terms were not included to aid interpretation of the first-order effects. In both models, vifs were < 5 for all variables. Model averaging was carried out as above.

Finally, to assess whether fungal alpha diversity was associated with differences in host survival, a generalised linear model with a binomial error structure and logit link function was constructed using *stats* 4.1.1 [63]. Survival to the next breeding season (1, yes; 0, no) was included as the dependent variable. Fungal alpha diversity, host sex, age, and territory quality were included as independent variables. Sample year (2017, 2018, or 2019) was included to control for the differences in survival probabilities between years [60]. Squared terms for age and alpha diversity were tested but were subsequently removed due to a lack of significance. This analysis was conducted using the full dataset (*n* = 259 samples; 37 individuals died, 218 survived to the following season). The significance of terms was assessed as above. Although the exact date of death is not known, for 25 of the individuals that died by the next breeding season, the point of GM sampling was the last time they were observed in the population. The remaining 12 individuals that died were observed (but not sampled) again in the same breeding season as GM sampling took place; in some cases, this was up to 7 weeks after their last GM sample was taken. There was also a median period of 4 months (range 3–7) between the point when GM samples were taken and when the population was next censused to assess survival. Thus, it is possible that some of the individuals were sampled several months before their point of death.

#### Beta diversity

The unrarefied reads (filtered to remove samples with < 5000 reads) were used in beta diversity analyses. ASVs were further filtered from the dataset if they were present in less than 1% of samples (approximately 3 samples) to remove exceptionally rare taxa that may disproportionately influence beta diversity metrics. There were 794 ASVs remaining across 259 samples after filtering. ASV abundances were transformed using the centred log ratio (CLR) transform function in *microbiome* 1.14.0 [86]. This produces values that are scale invariant (i.e. not influenced by differences in library size across samples) and controls for the compositional nature of microbiome datasets [87]. To assess whether the DNA extraction method produced consistent results for beta diversity measurements, pairwise Euclidean distances were calculated, using the CLR-transformed ASV abundances, between duplicate extractions of the same sample or between extractions of different samples. Distances were compared using the Kruskal–Wallis test. Extraction duplicates were then removed, as described for alpha diversity analysis. The four “floater” individuals were also removed, as described above.

To test whether fungal GM composition varied according to host and environmental variables, a permutational analysis of variance (PERMANOVA) was performed on a Euclidean distance matrix of CLR-transformed ASV abundances, using the *adonis2* function in *vegan* 2.5.7 [88, 89], with 9999 permutations. We first analysed the full dataset (*n* = 255 individuals) to maximise power. The host’s age, sex, heterozygosity (*H*_*s*_), breeding season (major or minor), territory quality, and time of sampling (AM or PM) were included in the analysis. Survival to the next breeding season (yes or no) was included as a factor in the model. The amount of time each sample was stored at 4 °C in the field was also controlled for in the analysis. The function *betadisper* was used to check for the homogeneity of group dispersion values [88, 89]. Differences in fungal GM composition between the groups were visualised using a principal components analysis (PCA). We further tested for associations between variables and shifts across specific principal component axes using the *envfit* function in *vegan* 2.5.7; this uses linear model permutations to map variables onto an ordination.

In the second analysis, the dataset was restricted to include individuals with complete MHC and *TLR3* genotype data, as described for alpha diversity analysis (*n* = 189 individuals). Two different models were run to test whether fungal GM composition was associated with immunogenetic variation. Firstly, a PERMANOVA was performed as described above for the full dataset, but with MHC-I and MHC-II diversities as additional predictor variables. The second PERMANOVA model instead included the presence/absence of each MHC allele as predictor variables, as described for alpha diversity analysis. *TLR3* genotype (*TLR3*^*AA*^, *TLR3*^*AC*^, or *TLR3*^*CC*^) was also included in both models. All other variables included in the full analysis were controlled for. Neither MHC diversity, nor the presence/absence of specific alleles correlated with survival to the next breeding season (*P* > 0.1 in GLMs), and so these terms could be included in the same PERMANOVA analysis.

To establish whether specific ASVs were differentially abundant across particular groups of individuals, an analysis of compositions of microbiomes with bias correction (ANCOM-BC) was carried out, using *ANCOMBC* 1.1.5 [90]. As part of ANCOM-BC, the Benjamini and Hochberg method was used to correct *P* values for multiple testing [91]. A cutoff of *P*_adj_ < 0.05 was used to assess significance.

## Results

### Characterising the fungal GM of the Seychelles warbler

Following quality filtering and the removal of samples with < 5000 reads, the number of high-quality fungal reads ranged from 5013 to 1,204,831 across the remaining 265 faecal samples. A total of 2540 fungal ASVs were detected across all samples, with a mean of 51.69 ± 27.90 (SD) ASVs inferred per sample. Comparisons of samples that had been extracted twice showed that the extraction method was consistent; pairwise distances were significantly lower between repeat extractions of the same sample, than between extractions from different samples (*P* < 0.05 in Kruskal–Wallis tests, Additional file 1: Fig. S2). This was the case for both observed ASV richness and fungal GM composition (Additional file 1: Fig. S2).

Following the removal of duplicate extractions and rarefaction to 5000 reads, 2530 fungal ASVs remained across 259 samples (mean ASV richness per sample = 49.49 ± 26.02 SD). The mean fungal Shannon diversity was 2.29 ± 0.66 across samples. On average, a greater number of bacterial ASVs were detected per sample than fungal ASVs (mean of 278.53 ± 167.77 SD per sample). The mean bacterial Shannon diversity was 3.86 ± 1.15. There was a significant, positive correlation between the fungal and bacterial ASV richness identified in each sample (*r* = 0.50, *P* < 0.001; Additional file 1: Fig S3). A significant, positive correlation was also identified between fungal and bacterial Shannon diversity measures (*r* = 0.18, *P* = 0.003; Additional file1: Fig S3).

**Fig. 1:**
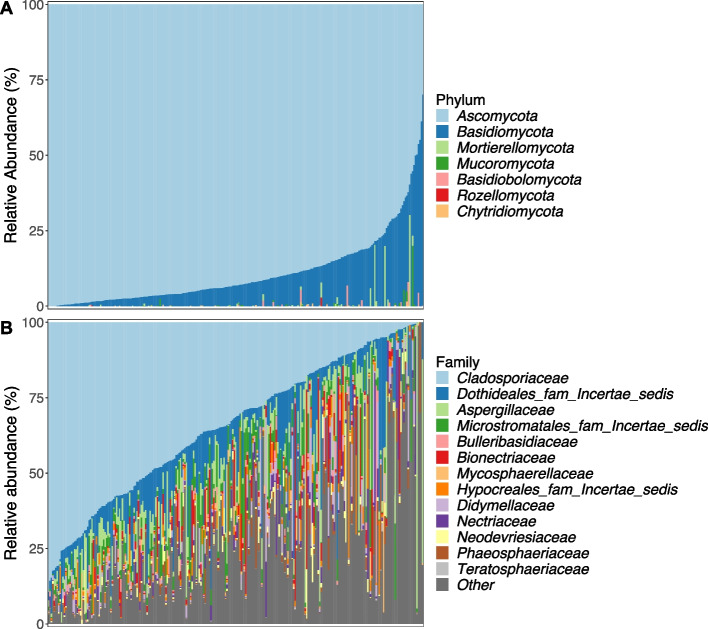
The relative abundance (%) of fungal **A** phyla and **B** families in Seychelles warbler faecal samples (*N* = 259 individuals). Each vertical bar represents a separate faecal sample. Samples are ordered according to the abundance of (a) *Ascomycota* and (b) *Cladosporiaceae*. Families with a median relative abundance of < 1% across samples are collapsed into the category “other”

A total of seven fungal phyla were detected across samples (Fig. 1a). The phylum *Ascomycota* was the most dominant and was present in 100% of samples with a mean relative abundance of 89.29% ± 11.69% (SD). This was followed by *Basidiomycota* which was present in 97% of samples with a mean abundance of 10.01% ± 11.07% (SD) across samples. All other phyla were present in fewer than 10% of samples. At lower taxonomic levels, the 2530 detected ASVs grouped into a total of 39 fungal classes, 249 families, and 475 genera.

Core fungal taxa were identified as those that were present in at least 50% of samples at a minimum relative abundance of 0.1%. A total of 13 core families were identified, four of which had a prevalence of over 80% (Additional file 1: Table S2). These were *Cladosporiaceae* (mean relative abundance across all samples 34.11% ± 23.29% SD), *Dothideales incertae sedis* (10.79% ± 11.96%), *Aspergillaceae* (7.43% ± 12.72%), and *Bionectriaceae* (3.48% ± 8.56%) (Additional file 1: Table S2). A total of 13 core genera were also identified, three of which were present in more than 80% of samples: *Cladosporium* (34.03% ± 23.27%), *Hortaea* (10.79% ± 11.95%), and *Penicillium* (3.79% ± 8.78%) (Additional file 1: Table S2). Further to this, a total of 7 core ASVs were identified and classified as the following at species level: *Cladosporium dominicanum* (28.57% ± 20.77%), *Hortaea werneckii* (28.57% ± 20.77%), *Cladosporium coloradense* (3.06% ± 5.9%), *Penicillium citrinum* (2.13% ± 6.31%), an unidentified species in the family *Didymellaceae* (2.19% ± 5.38%), and two ASVs identified as *Sympodiomycopsis kandeliae* (1.96% ± 3.03% and 1.23% ± 2.55%) (Additional file 1: Table S2). Despite the presence of shared taxa, the composition and diversity of the fungal GM varied substantially across individuals in the population (Fig. 1).

### The impact of host and environmental variables on fungal GM alpha diversity

**Fig. 2:**
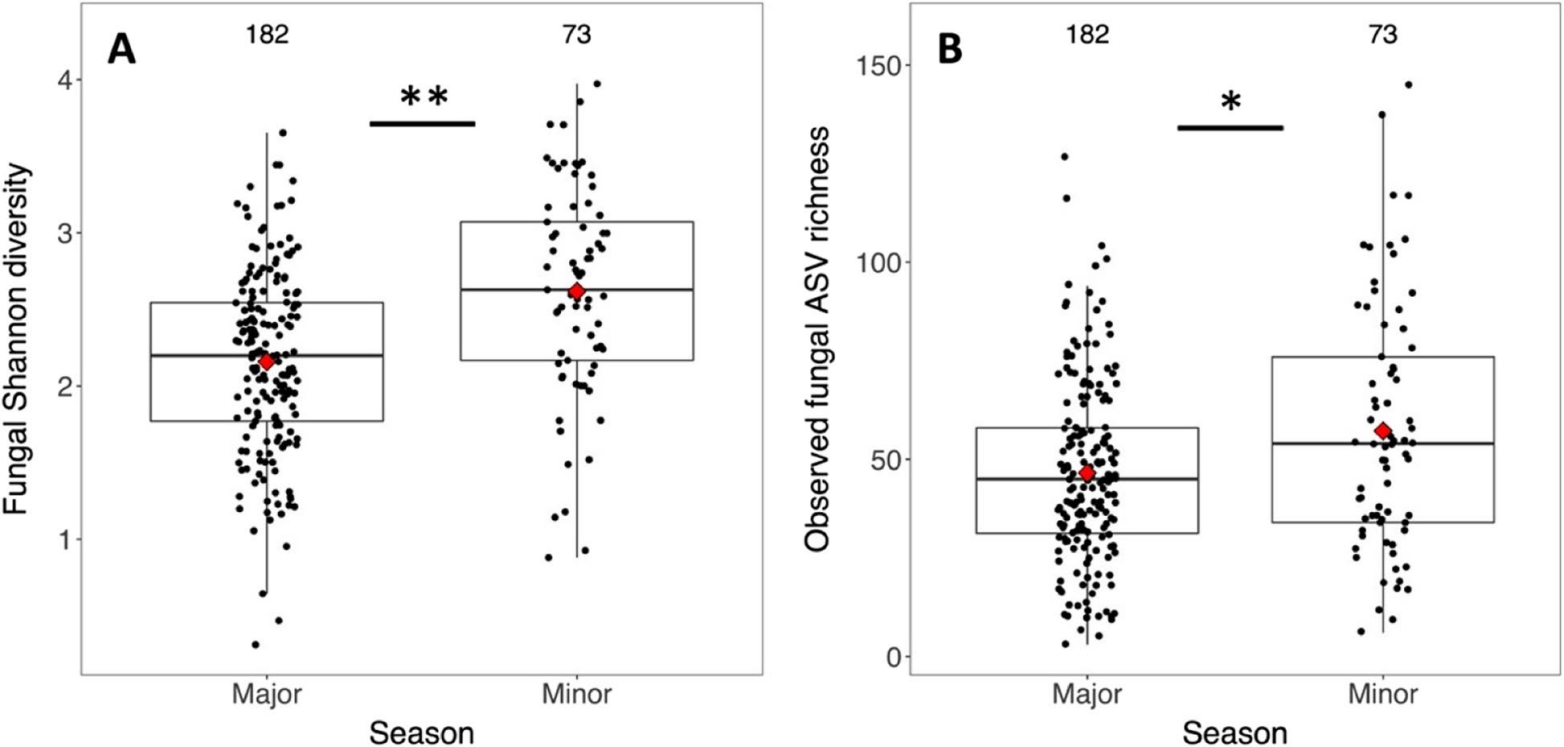
The impact of season on fungal **A** Shannon diversity and **B** ASV richness in the gut microbiome of the Seychelles warbler (*N* = 255 individuals). Major season = 182 samples, minor season = 73 samples. Black points represent the raw data. Boxes span the interquartile (25–75%) range. The median is marked by a horizontal line, and the mean is marked by a red diamond. Whiskers extend to 1.5 times the interquartile range. **P < 0.001, *P < 0.01 from a linear model

We first analysed the full dataset (*n* = 255 individuals) to establish whether fungal GM alpha diversity varied according to host and/or environmental factors, before testing for the effects of immunogenetic variables using a reduced dataset. Fungal alpha diversity was significantly higher in the minor versus the major breeding season (Table 1, Fig. 2). However, neither territory quality nor time of sampling was significantly associated with fungal alpha diversity (Table 1). Host age, sex, and genome-wide heterozygosity were also not associated with fungal alpha diversity (Table 1). The length of time that samples were stored at 4 °C had no effect on fungal Shannon diversity (Table 1), but there was a weak, negative association between storage time at 4 °C and the fungal ASV richness detected in samples (Table 1). As such, storage time at 4 °C was controlled for in all downstream analyses.

**Table 1:** The impact of host and environmental factors on fungal alpha diversity in the gut microbiome of the Seychelles warbler (*n* = 255 individuals). (A) Shannon diversity and (B) observed ASV richness were used as the response variable in two separate models. Estimates and standard errors (SE) are based on linear conditional model-averaged estimates. Significant (*P* < 0.05) predictors are shown in bold. The reference categories for categorical variables were as follows: female (sex), major (season), and AM (time of day)

Predictor	Estimate	SE	*z*	*P*
**A) Shannon diversity**				
**Intercept**	**2.185**	**0.063**	**34.597**	** < 0.001**
Age	− 0.050	0.079	0.633	0.527
Sex (male)	0.044	0.078	0.555	0.579
Heterozygosity	− 0.073	0.078	0.932	0.351
**Season (minor)**	**0.453**	**0.089**	**5.089**	** < 0.001**
Territory quality	− 0.059	0.080	0.736	0.462
Time of day (PM)	− 0.131	0.080	1.642	0.101
Time stored at 4 °C	0.044	0.082	0.527	0.599
**B) Observed ASV richness**				
**Intercept**	**3.855**	**0.055**	**69.679**	** < 0.001**
Age	− 0.047	0.068	0.688	0.491
Sex (male)	− 0.027	0.067	0.402	0.687
Heterozygosity	− 0.059	0.067	0.872	0.383
**Season (minor)**	**0.262**	**0.081**	**3.222**	**0.001**
Territory quality	− 0.025	0.070	0.362	0.717
Time of day (PM)	− 0.120	0.068	1.754	0.079
**Time stored at 4 °C**	** − 0.161**	**0.070**	**2.284**	**0.022**

### The effect of immunogenetic variation on fungal GM alpha diversity

**Fig. 3:**
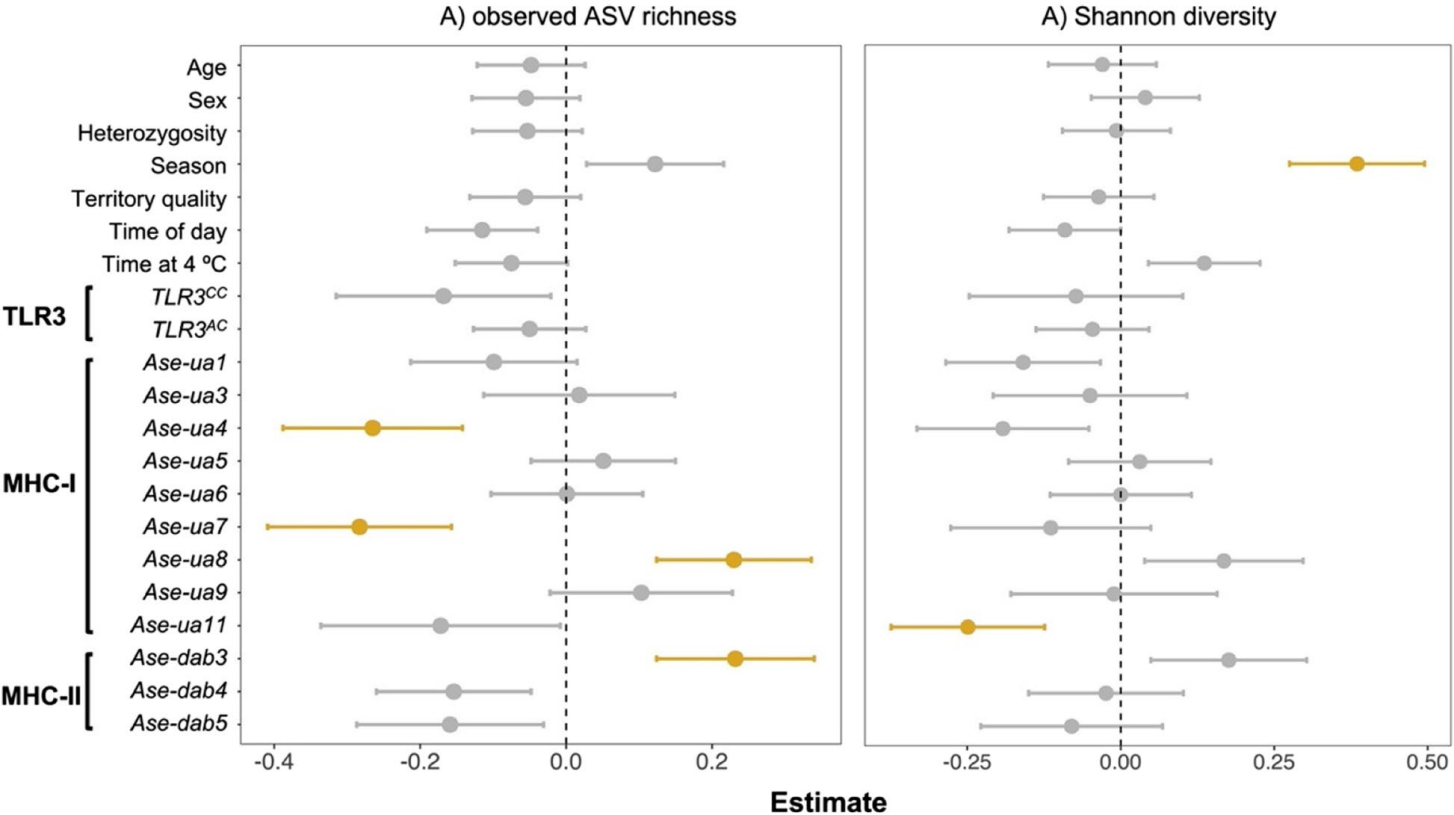
The effect of MHC alleles and *TLR3* genotype on fungal alpha diversity in the gut microbiome of the Seychelles warbler, controlling for other host and environmental variables. Diversity was measured as **A** observed ASV richness and **B** Shannon diversity. *N* = 189 individuals. Estimates and standard errors are based on linear conditional model-averaged estimates. An estimate of greater or less than zero indicates increased or reduced alpha diversity, respectively. Significant terms (*P* < 0.05) are highlighted in yellow. Reference categories for categorical variables were as follows: absent (for all MHC alleles), *TLR3*.^*AA*^ (TLR3 genotype), female (sex), major (season), and AM (time of day)

There was no relationship between individual MHC diversity and fungal GM alpha diversity (when measured as either Shannon diversity or observed ASV richness; Additional file 1: Table S3). *TLR3* genotype also had no significant effect on GM alpha diversity (Additional file 1: Table S3). In contrast, the presence of three MHC-1 alleles and one MHC-II allele was significantly associated with changes in ASV richness (Fig. 3). Individuals that carried the MHC-I allele *Ase-ua4* or *Ase-ua7* had significantly lower ASV richness compared to individuals without these alleles (Fig. 3, Additional file 1: Table S4 and Fig. S4a,b)*.* In contrast, individuals that carried the MHC-I allele *Ase-ua8* had greater fungal ASV richness compared to individuals without this allele (Fig. 3, Additional file 1: Table S4 and Fig. S4c). This was also the case for individuals possessing the MHC-II allele *Ase-dab3* (Fig. 3, Additional file 1: Table S4 and Fig. S4d)*.* The mean number of ASVs (± SD) detected in samples when *Ase-ua4* was absent was 50.34 ± 24.73, compared to 48.91 ± 24.52 when the allele was present (Additional file 1: Fig. S4). In contrast, changes were considerably greater for the allele *Ase-ua7*; a mean of 57.96 ± 26.81 ASVs was detected when the allele was absent compared to 47.61 ± 24.51 when present (c.a. a 20% loss in ASV richness). The same was also true for the alleles *Ase-ua8* and *Ase-dab3* (*Ase-ua8*: 48.59 ± 24.81 absent, 55.97 ± 27.15 present; *Ase-dab3* 47.22 ± 21.35 absent, 57.80 ± 33.06 present). However, none of these alleles was significantly associated with shifts in Shannon diversity (Fig. 3, Additional file 1: Table S4). Instead, there was a marginally significant, negative relationship between the presence of *Ase-ua11* and Shannon diversity (Fig. 3, Additional file 1: Table S4).

### The impact of host and environmental variables on fungal GM composition

**Fig. 4:**
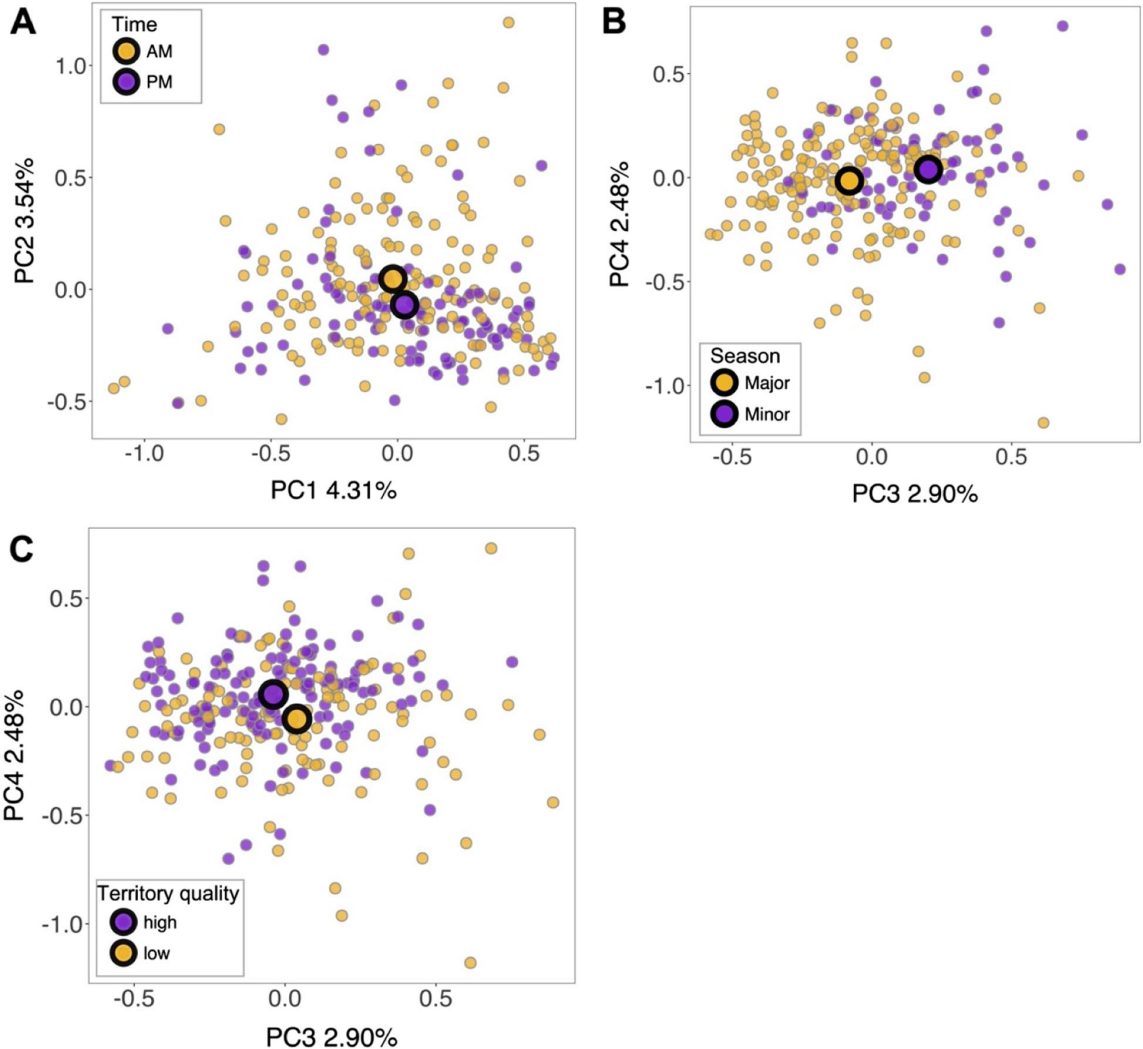
Shifts in fungal gut microbiome composition according to **A** time of day, **B** sampling season, and **C** territory quality in the Seychelles warbler. PCA ordination was carried out using Euclidean distances calculated using centred log ratio (CLR)-transformed amplicon sequencing variant (ASV) abundances. Each point represents a unique gut microbiome sample (*N* = 255). Large circles represent the group centroids. For clarity, samples were grouped into discrete categories for territory quality (high or low depending on whether it was above or below the median territory quality of 12,678, respectively). Principal components 1, 2, 3, and 4 explained 4.31%, 3.54%, 2.90%, and 2.48% of the variation in gut microbiome structure, respectively

A PERMANOVA analysis using the full dataset revealed that fungal GM composition differed significantly between the major and minor breeding seasons (Table 2, Fig. 4); fungal GM composition was also more variable in the minor breeding season (*betadisper* test: *F*_1,253_ = 19.654, *P* < 0.001, Additional file 1: Fig. S5). Shifts in GM composition were additionally associated with differences in territory quality (Table 2, Fig. 4) and, weakly, with the time of day that an individual was sampled (Table 2, Fig. 4). GM composition also varied significantly according to the amount of time samples were stored at 4 °C (Table 2) indicating the importance of controlling for sample storage methods. Differences in host age, sex, and genome-wide heterozygosity were not associated with shifts in GM composition (Table 2).

**Table 2:** PERMANOVA analysis of fungal gut microbiome communities in the Seychelles warbler. Euclidean distances were calculated based on centred log ratio (CLR)-transformed amplicon sequencing variant (ASV) abundances. Significant predictors (*P* < 0.05) are shown in bold. The analysis included 255 individuals

Predictor	df	*R*^2^	*F*	*P*
Age	1	0.004	1.148	0.115
Sex (male/female)	1	0.004	0.947	0.658
Heterozygosity	1	0.004	1.081	0.219
**Season (minor/major)**	**1**	**0.012**	**3.200**	** < 0.001**
**Territory quality**	**1**	**0.006**	**1.466**	**0.003**
**Time of day (AM/PM)**	**1**	**0.005**	**1.267**	**0.020**
**Time stored at 4 °C**	**1**	**0.007**	**1.928**	** < 0.001**
**Survival (yes/no)**	**1**	**0.005**	**1.302**	**0.019**

Points clustered on a PCA ordination according to significant environmental variables (Fig. 4); clustering along the first two principal component axes was significantly associated with the time of day (envfit model: *R*^2^ = 0.018, *P* = 0.010, Fig. 4a) and sampling season (*R*^2^ = 0.064, *P* < 0.001), although points clustered more strongly according to season along axes three and four (*R*^2^ = 0.106, *P* < 0.001, Fig. 4b). Placement of points along the third and fourth axes was also influenced by differences in territory quality (*R*^2^ = 0.048, *P* = 0.003, Fig. 4c).

Differential abundance tests revealed that 31 ASVs were significantly differentially abundant between the major and minor breeding seasons (*P* < 0.05 in an ANCOM-BC test; Additional File 1: Fig. S6 and Additional file 2). Of these, seven were more abundant in the major season. These were in the fungal orders *Capnodiales* (two ASVs in the genus *Cladosporium* and one ASV in the genus *Recurvomyces*) and *Eurotiales* (three ASVs in the genus *Penicillium*, one in the genus *Aspergillus*). The remaining 24 ASVs, belonging to ten identified fungal orders, were significantly more abundant in the minor breeding season (Additional File 1: Fig. S6 and Additional file 2). The fungal orders *Pleosporales* (10 ASVs) and the *Hypocreales* (three ASVs) contributed over 50% of these ASVs.

There were seven ASVs that were differentially abundant between high and low territory qualities (see Additional file 2 for a full taxonomic breakdown). Quality scores above or below the median territory quality (12,678) were classified as high or low, respectively, in this analysis. Six of the ASVs were more abundant in high territory qualities (Additional file 2); five of these were in the fungal phylum *Basidiomycota* and belonged to two orders, namely, *Tremallales* (one ASV in the genus *Dioszegia* and one in an unidentified genus) and *Microstromatales* (two ASVs in the genus *Sympodiomycopsis *and one in the genus *Jaminaea*, respectively). The other ASV was in the phylum *Ascomycota* (an unidentified member of the family *Didymellaceae*). One ASV in the phylum *Ascomycota* was more abundant in low-quality territories (Additional file 2); this was in the order *Capnodiales* (unidentified below order level). Differential abundance testing did not identify any ASVs that were significantly differentially abundant between samples taken in the morning or afternoon.

### The impact of immunogenetic variation on fungal GM composition

**Fig. 5:**
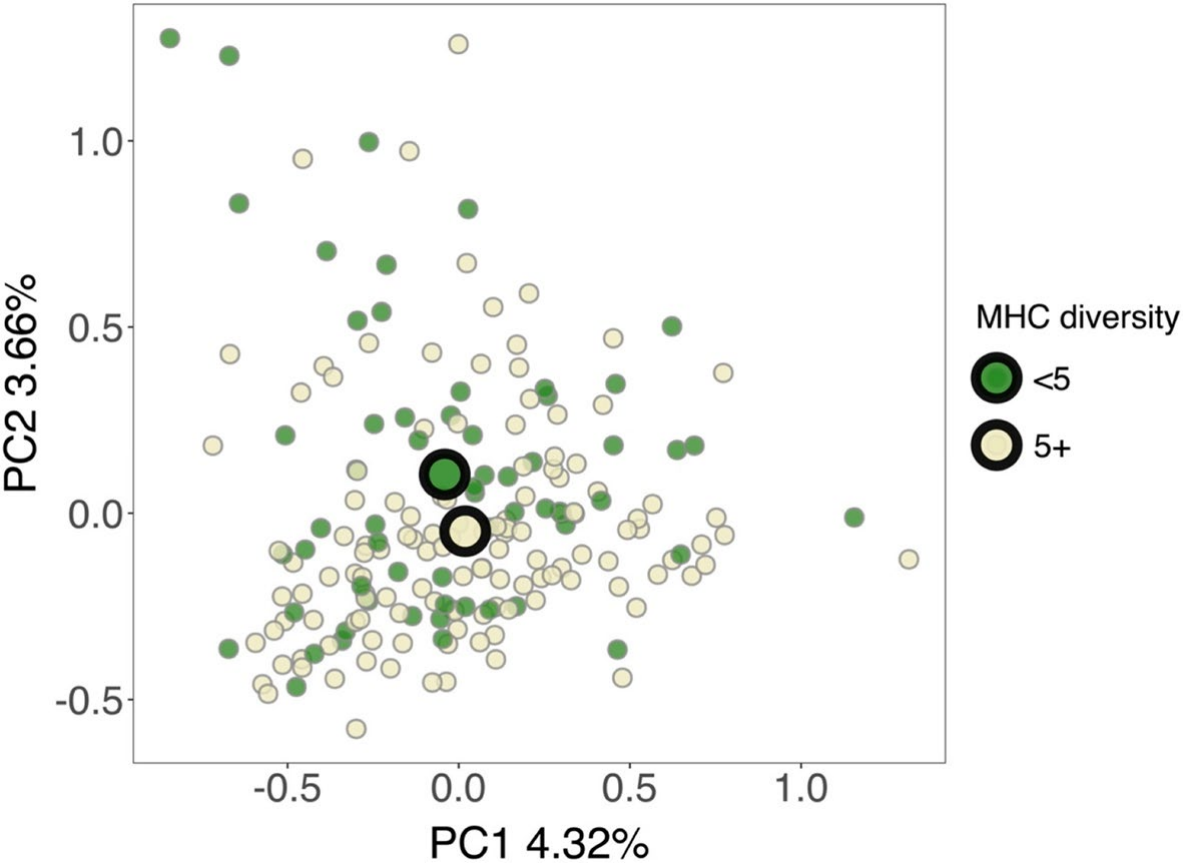
Shifts in fungal gut microbiome (GM) beta diversity according to MHC-I diversity in the Seychelles warbler. Principal components analysis (PCA) was carried out using Euclidean distances based on CLR-transformed abundances of amplicon sequencing variants (ASVs). Principal components 1 and 2 explained 4.32% and 3.66% of the variation in GM community structure, respectively. *N* = 189 individuals were included in the analysis. For clarity, individuals were grouped into discrete categories, according to whether they had 2–4 (< 5, green points) or 5–7 (5 + , yellow points) alleles. Large circles represent group centroids

A PERMANOVA analysis using the immunogenetic dataset demonstrated that there was a marginally significant association between MHC-I diversity and fungal GM composition (Table 3), but MHC diversity was not associated with differences in GM variability (*betadisper* test: *F*_1,187_ = 1.018, *P* = 0.405). MHC-I diversity was associated with the placement of points along the first two axes of a PCA ordination (*envfit* model: *R*^2^ = 0.044, *P* = 0.015; Fig. 5). Testing identified two ASVs that were differentially abundant across individuals with low (2–4 alleles) or high (5–7 alleles) MHC-I diversity (*P* < 0.05 in an ANCOM-BC test; Additional file 2). One ASV was more abundant in individuals that had low diversity; this was in the order *Botryosphaeriales* (genus *Lasiodiplodia*). The second ASV was more abundant in individuals that had high MHC-I diversity; this was in the order *Saccharomycetales* (genus *Sympodiomyces*). MHC-II diversity and *TLR3* genotype had no effect on GM composition (Table 3). A separate PERMANOVA analysis showed that the presence/absence of specific MHC-I or MHC-II alleles was also not associated with variation in fungal GM composition (Additional file 1: Table S5).

**Table 3:** The influence of MHC diversity on fungal gut microbiome beta diversity in the Seychelles warbler. Based on a PERMANOVA analysis of Euclidean distances, calculated using centred log ratio (CLR)-transformed amplicon sequencing variant (ASV) abundances. Significant predictors (*P* < 0.05) are shown in bold. The analysis included 189 individuals

Predictor	df	*R*^2^	*F*	*P*
Age	1	0.006	1.078	0.242
Sex (male or female)	1	0.005	1.002	0.456
Heterozygosity	1	0.005	1.014	0.417
**Season (minor or major)**	**1**	**0.011**	**2.178**	** < 0.001**
**Territory quality**	**1**	**0.008**	**1.439**	**0.005**
Time of day (AM or PM)	1	0.005	1.023	0.378
**Time stored at 4 °C**	**1**	**0.009**	**1.761**	** < 0.001**
**Survival**	**1**	**0.007**	**1.412**	**0.006**
*TLR3* genotype (*AA*, *AC*, or *CC*)	2	0.010	0.987	0.531
**MHC-I diversity**	**1**	**0.006**	**1.232**	**0.036**
MHC-II diversity	1	0.005	0.912	0.769

### Fungal GM structure and host survival

**Fig. 6:**
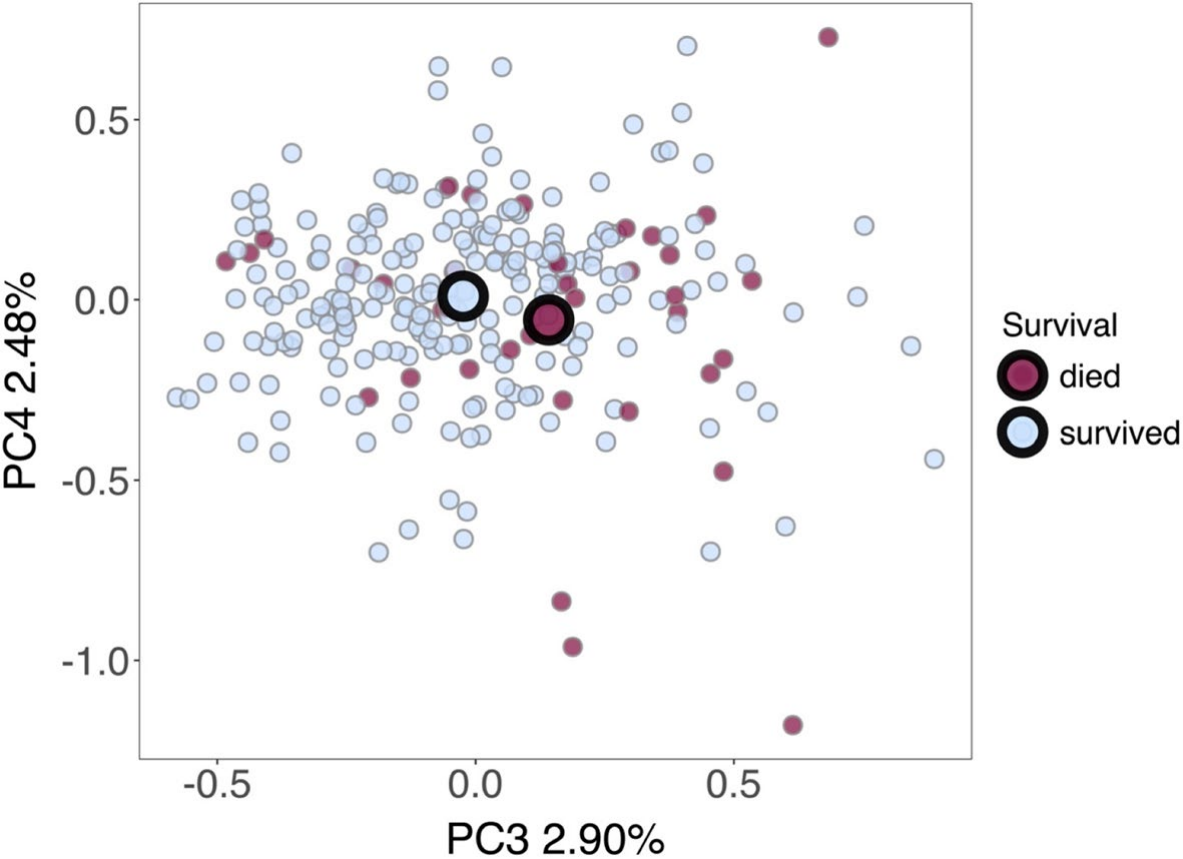
The association between fungal gut microbiome (GM) structure and differential survival in the Seychelles warbler. Principal components analysis (PCA) was carried out using Euclidean distances based on CLR-transformed abundances of amplicon sequencing variants (ASVs). Points are coloured according to whether individuals survived (blue) or died (red) by the next breeding season. Principal components 3 and 4 explained 2.90% and 2.48% of the variation in GM community structure, respectively. *N* = 255 individuals were included in the analysis (218 individuals survived, 37 individuals died). Large circles represent group centroids

There was no significant association between fungal GM alpha diversity and the probability that an individual survived to the next breeding season (Additional file 1: Table S6) for either the Shannon diversity or observed ASV richness metric. However, survival to the next breeding season was significantly associated with shifts in fungal GM composition in a PERMANOVA analysis (Table 2, Fig. 6). Points predominantly separated along the PC3 and PC4 axis of a PCA ordination according to survival (envfit model: *R*^2^ = 0.025, *P* = 0.002, Fig. 6). A *betadisper* test indicated that there was no difference in fungal GM variability between individuals that died and those that survived by the following breeding season (*F*_1,253_ = 0.013, *P* = 0.910).

Differential abundance testing indicated that six fungal ASVs were more abundant in individuals that survived to the following breeding season, compared to those that died (*P* < 0.05 in an ANCOM-BC test, Additional file 2). Three of these belonged to the fungal class *Sodariomycetes*; these were members of the genera *Gliomastix*, *Chordomyces*, and *Acrostalagmus*. A further two ASVs belonged to the order *Microstromatales* (both in the genus *Sympodiomycopsis*). The remaining ASV belonged to the order *Venturiales* (genus *Ochroconis*). No ASVs were identified that were significantly more abundant in individuals that died.

## Discussion

In this study, we characterised variation in the fungal GM in a natural population of the Seychelles warbler. We then investigated the extent to which this variation was associated with environmental and host factors and, importantly, subsequent host survival. Variation in fungal GM composition was associated with breeding season, territory quality, and the time of day at which an individual was sampled. In contrast, there were few host variables associated with fungal GM variation. Neither host age, sex, genome-wide heterozygosity, or *TLR3* genotype were associated with differences in fungal GM structure. However, the presence of three MHC-I alleles and one MHC-II allele was associated with changes in fungal ASV richness, whilst the presence of a different MHC-I allele was associated with a reduction in Shannon diversity. Despite these associations, differences in diversity based on allele presence were reasonably small and did not scale up to dissimilarities in GM composition. However, overall MHC-I allelic diversity was (to a limited extent) associated with fungal GM composition. Finally, variation in fungal GM composition was associated with differential survival, and the abundances of specific fungal ASVs differed between individuals that survived versus those that died by the following breeding season.

### The fungal GM of the Seychelles warbler

The composition of the Seychelles warbler fungal GM was similar to that of other vertebrate taxa, including passerine species, in that it was dominated by the phyla *Ascomycota* and *Basidiomycota* [92]. Despite substantial variation in fungal GM structure across individuals in the Cousin Island population, we identified several core taxa that were shared across the majority (> 50%) of individuals. Several of these taxa are common members of the vertebrate GM. For example, members of the family *Aspergillaceae* (including *Aspergillus* and *Penicillium* species) are often identified in GM samples taken from human and non-human primates [14, 93–95]. The genus *Cladosporium* is one of the most dominant filamentous fungal taxa in the human GM [93, 96, 97] and is abundant in faecal samples taken from other vertebrate species [94, 98]. This genus is also more abundant in the GM of insectivorous, versus phytophagous, bat species suggesting that there may be some association between its abundance and having an insect-based diet [98].

It is likely that many of the core taxa identified in the Seychelles warbler GM are environmentally acquired. For example, *Cladosporium* species are widely distributed in the environment and have been isolated from a diverse range of habitats including soils, plant material and insects [99]. Similarly, species in the genus *Sympodiomycopsis*, have previously been isolated from insects [100] and mangrove plants [101]. Thus, Seychelles warblers may acquire such species by ingesting their insect prey. Since birds possess relatively short intestinal tracts as an adaptation to improve flight efficiency, they may be particularly prone to acquiring microbial strains from their environment. Thus, these species may only transiently pass through the gut ecosystem before being excreted in faecal matter [102–104].

Differentiating between resident gut commensals and transient colonisers is challenging given our limited understanding of host-microbe relationships within the intestinal tract and the fact that many fungal strains remain poorly characterised [13, 105, 106]. Furthermore, the extent to which transient strains can elicit changes in the resident gut microbiota and perform functions relevant to the host remains unclear [105, 107, 108]. In future studies, it will be important to establish the degree to which different fungal species consistently colonise their host and the functionality of these strains within the gut ecosystem [105, 106]. Although such studies may require experimental manipulation, longitudinal sampling of wild individuals could help to determine which microbial taxa are stably acquired, and metagenomic sequencing would provide further information on their functionality.

The selection of primers for fungal community analysis remains a subject of much debate since primer choice can significantly influence community composition [72, 109, 110]. The specific ITS2 primers used in this study were chosen as they provide superior coverage compared to other ITS2 primers (by amplifying > 90% of fungal groups) and those targeting the ITS1 region which, in comparative studies, have been shown to introduce greater taxonomic bias and underestimate levels of fungal diversity [72, 111]. The ITS2 region can also provide greater taxonomic resolution than rRNA markers (such as the 18S subunit) [72, 110]; this is likely to be important when assessing inter-individual variation at a fine scale. However, we acknowledge that 18S amplicons can be useful for calculating measures of phylogenetic diversity given the possibility of sequence alignment across a broad range of fungal taxa [72]. The primers used in this study were shown to accurately capture two mock community controls of varying complexity. However, biases could still exist, and combining different primer sets may allow fungal communities to be captured with further accuracy in the future.

### Environmental drivers of fungal GM variation

Season explained the largest proportion of variance in fungal community composition across sampled individuals. Fungal GM alpha diversity and variation in GM composition were also greater in the minor, compared to the major, breeding season. Climatic variables can vary considerably between seasons on Cousin Island, for example, rainfall tends to be greater in the minor season [58]. Such variation likely impacts microbial communities in the external environment [112] meaning that hosts are exposed to different fungal species either directly or indirectly via their insect prey. Differing weather conditions could also have an impact on island-wide insect availability which, by altering the type or quantity of diet available, could influence host condition and/or conditions within the intestinal tract and, in turn, the fungal GM [113]. Indeed, previous research has shown that island-wide insect availability tends to be significantly greater during the major season on Cousin Island [114]. A similar effect of season on fungal GM communities has been observed in wild Tibetan Macaques (*Macaca thibetana*), in which alpha diversity and certain fungal taxa increased in winter months; these differences were thought to be driven by changing food availability and dietary components [14].

The reproductive behaviour of the warblers also changes notably across the year, with the majority of birds breeding in the major season [58]. As such, changes in fungal GM structure between seasons could also be driven, in part, by physiological or behavioural changes related to reproduction. Relationships between breeding activity (e.g. hormonal changes, nest building, and copulation) and the microbiota have been identified when studying the bacterial GM in other bird species [11, 115, 116]. Several of the fungal taxa that increased in abundance in the GM of Seychelles warblers sampled in the major season are known to be plant-associated and thus could have been acquired from nesting material. For example, *Cladosporium* species have been isolated from mangrove plants in tropical regions [117], and species in the genus *Penicillium* are known to live on, and decompose, plant material [118, 119].

Fungal GM composition also differed according to the quality of the host’s territory. As with seasonal differences, this relationship could arise due to environmental filtering, whereby the environmental conditions within a particular territory determine the range of fungal species that can proliferate and colonise the host [120, 121]. Thus, birds inhabiting territories that are exposed to differing levels of foliage cover, humidity and/or salt spray, may come into contact with different fungal taxa. However, variation in territory quality has also been associated with differences in host condition in the Seychelles warbler [122, 123]. Experimental manipulations and studies of wild animals have shown that prolonged stress, differing food availability, and host infection status can all have an impact on the GM, including the composition of fungal communities [124–126]. Thus, physiological differences associated with varying territory qualities may have consequences for fungi in the intestinal tract.

### Host genetics and fungal GM variation

Compared to environmental variables, host genetic factors appeared to have a weaker influence on fungal GM structure. Genome-wide heterozygosity was not associated with shifts in GM composition across individuals. This is surprising given that heterozygosity is a measure of inbreeding. Inbreeding occurs frequently in the Seychelles warbler, and previous studies have identified an effect of inbreeding depression on fitness in this population [127–129]. Thus, it might be expected that, via an effect on host physiology and condition, heterozygosity would also be associated with GM differences. However, some of the costs of inbreeding have been found to be environmentally dependent in this species, whereby they are only observed under very poor conditions [127–129]. Thus, further years of data collection, which incorporate temporal variation in environmental conditions, may be needed to identify the effects of inbreeding on the GM.

It is perhaps not surprising that *TLR3* variation had no influence on the fungal GM, since it encodes receptor molecules that bind to viral dsRNA [53]. It is possible that polymorphisms at other TLR loci may have a greater influence on fungal GM variation in wild populations. For example, molecules encoded by the *TLR4* gene have been shown to specifically detect fungal peptides [19, 130]. However, the majority of TLR loci (including *TLR4*) lack variation in the Seychelles warbler [52].

The presence of two MHC alleles was associated with a significant reduction in fungal ASV richness (*Ase-ua7* and *Ase-ua4*), whilst one MHC-I allele (*Ase-ua8*) and one MHC-II allele (*Ase-dab3*) were associated with an increase in ASV richness. However, these effects were limited; the mean number of ASVs that were lost or gained ranged from between two and ten ASVs, though in the latter case, this accounted for c.a. 20% of fungal ASVs carried by an individual. There was also no association between the presence of these alleles and Shannon diversity, suggesting that only a small number of rare ASVs may have been gained or selectively eliminated, rather than these alleles having a wider influence on taxon abundances and community evenness. Only one different allele (*Ase-ua11*) had a marginally significant influence on Shannon diversity. However, the presence/absence of these individual alleles did not have a significant influence on overall fungal GM composition. Such small effects are not unprecedented; a study on the bacterial GM of wild mouse lemurs (*Microcebus griseorufus*) found that the majority of MHC-I and MHC-II supertypes influenced the abundance of a very small number of ASVs (between one and three taxa), rather than having an overarching effect on the entire GM community [41]. This also concurs with an experimental study on laboratory mice (*Mus musculus*) which found that MHC genotype had a very limited effect on the GM; this study concluded that the immune activity of the MHC may be reserved for a small number of specific colonisers such as pathogens, or intrusive GM commensals [131]. More generally, small effect sizes may be expected when investigating relationships between MHC variation and immunocompetence, given that the MHC plays only a small part in a complex network of immune pathways [132].

The diversity of MHC-I alleles (but not MHC-II alleles) was associated with shifts in GM community composition. This is despite that fact that none of the MHC-I alleles was individually associated with significant compositional shifts. However, the association between MHC-I diversity and GM composition was relatively weak, explaining 0.6% of the variance in GM community structure. Furthermore, only two ASVs were identified as being differentially abundant between individuals with high and low MHC-I diversity; again, this is consistent with the idea that the MHC may act to target specific pathogens or tolerate specific commensal strains, rather than having pervasive effects on the whole GM [41, 131]. High MHC diversity was associated with a reduction in the abundance of an ASV in the fungal family *Botryosphaeriaceae*. Members of the *Botryosphaeriaceae* have predominantly been found in association with plants, often as pathogens [133], but certain genera can cause opportunistic infections in animal species [134]. Thus, it is possible that this species was more likely to be detected and selectively eliminated from the GM of individuals carrying a greater repertoire of MHC-I motifs. The second differentially abundant ASV—a member of the genus *Sympodiomyces* (order *Saccharomycetales*)—was more abundant in the GM of individuals with high MHC-I diversity. Although little is known about the *Sympodiomyces*, members of the *Saccharomycetales* are common in adult human faeces [135] and may play a beneficial role in maintaining host metabolic health [136, 137]. However, further research is needed to characterise the function of these ASVs in the avian GM to fully understand the observed relationships.

Similar to other studies on the bacterial GM, MHC-I alleles appeared to have a larger influence on the fungal GM than MHC-II alleles [4, 41]. The possible mechanism underlying this pattern remains unclear, particularly as MHC-I receptors primarily interact with intracellularly derived peptides. However, it is possible that MHC-I diversity affects the fungal GM indirectly by influencing host conditions and/or altering susceptibility to other infectious agents. The MHC-I allele *Ase-ua4*, which was associated with a reduction in fungal ASV richness in this study, has previously been associated with differential survival in the Seychelles warbler [50]. Although the agents underlying this interaction are unknown, it is possible that they also influence the fungal GM. Another important consideration is that linkage disequilibrium between the MHC and genes that regulate other aspects of immunity and/or host physiology could also generate correlations between MHC-I alleles and the fungal GM. This could be particularly important in the Seychelles warbler, where considerable linkage disequilibrium may exist because of a recent bottleneck [138].

One caveat of our study is that MHC diversity was calculated as the number of distinct sequences detected at MHC loci. However, this number can be a poor predictor of functional diversity [139]. Indeed, several studies on wild populations have shown that the degree of sequence divergence between MHC alleles is a stronger predictor of bacterial GM dissimilarity than the number of MHC motifs [40, 41]. Thus, sequence divergence between MHC-I alleles identified in the Seychelles warbler may be more strongly associated with shifts in GM composition and should be an avenue for future research.

Overall, the effect sizes linking immunogenetic variation to GM differences were smaller than those observed in a recent investigation of the bacterial component of the Seychelles warbler GM [4]. In the latter study, three MHC-I alleles and one MHC-II allele were associated with a reduction in bacterial GM alpha diversity, and four MHC-I alleles were associated with shifts in bacterial GM composition [4]. Out of those associated with changes in bacterial GM structure, the presence of three of the same alleles (*Ase-ua4*, *Ase-ua7*, and *Ase-ua11*) were also found to be associated with significant, but small, reductions in fungal alpha diversity in the present study. It is possible that at least some of the observed changes in fungal GM diversity were driven by cross-kingdom microbial interactions. It is well documented that bacterial taxa can promote or antagonise the growth of fungal species (and vice versa) and so changes in one community may influence both the structure and stability of the other [108, 140]. Thus, any impact of the MHC on bacterial communities may have downstream consequences for fungal components of the GM. Indeed, a positive correlation was found between the diversity of bacterial and fungal communities in Seychelles warbler GM samples. Although establishing the mechanisms underlying these relationships is beyond the scope of the current paper, investigating the interactions between different components of the GM will be a key area for future research.

Other host factors (age and sex) were not associated with differences in fungal GM structure. This is despite the fact that age was found to be a predictor of the bacterial component of the Seychelles warbler GM [4, 9] and has also been shown to influence the fungal GM of Tibetan macaques [14]. Our study did not incorporate chicks because of a low sample size, and so, it is possible that fungal GM communities differ in very early development, before becoming largely determined by the environment. However, future studies with longitudinal data for individuals will be needed to investigate the relationship between age and fungal GM structure in more detail. Host sex was also not associated with variation in the fungal GM. As male and female Seychelles warblers exhibit low levels of behavioural and morphological dimorphism, it may be that they are exposed to, and maintain, very similar fungal communities.

### Fungal GM and survival

In our study, variation in fungal GM composition (although not alpha diversity) was associated with differential survival. Six fungal ASVs were more abundant in individuals that survived to the next breeding season; however, no ASVs were significantly more abundant in those that died before the next season, suggesting that mortality may not be driven by the proliferation of pathogenic fungal species. It is likely that some of the differences in fungal composition between individuals that survived and those that died are correlated with environmental factors resulting in differences in host condition. Several of the ASVs that were more abundant in individuals that survived (e.g. those in the genus *Sympodiomycopsis*) were also found to be more abundant in individuals inhabiting better-quality territories in which insect food is more abundant. Thus, differences in fungal ASV abundances could be the result of differing host condition relating to factors such as food availability, stress, and/or because of differential exposure to fungal taxa in territories associated with higher or lower survival. Another possibility is that relationships between fungal taxa and survival arise due to changing microbial interactions within the GM. A previous study on the bacterial component of the Seychelles warbler showed that GM composition and the abundances of some pathogenic bacteria (e.g. *Mycobacterium* species) differed between adult individuals that survived or died by the next breeding season [9]. Thus, changes in fungal abundances could also represent a response to changes in bacterial communities, either due to direct microbe-microbe interactions or because abiotic conditions are altered in the intestinal tract.

Subsequent survival explained a small proportion of the overall variance in fungal GM composition (0.5% of the variation in the larger dataset). One limitation of our dataset is that some birds may have been sampled up to several months prior to death, when only small differences in the GM may have been detectable. For the majority of birds that died (25 out of 37 individuals) the point of GM sampling was the last time they were observed in the population. However, 12 individuals were seen again following GM sampling; in some cases, this was up to 7 weeks later, suggesting individuals had remained alive for a significant period post-sampling. Furthermore, there was a median period of 4 months between the last GM sample from these individuals and the next population census; individuals could have died at any point during this period. A study on captive juvenile ostriches (*Struthio camelus*) found that relationships between bacterial GM diversity and survival probability became stronger in the weeks directly leading up to death [10]. Thus, the fungal GM of Seychelles warblers may become more distinct closer to the point of death, as pathogenic strains proliferate and/or host condition declines. Further work, including the collection of longitudinal samples, is needed to understand if this is the case and to unpick the extent to which changes in the GM are the cause of death or if they simply represent a response to declining host health. Such studies will be crucial to understanding the extent to which the GM influences host fitness and evolutionary processes in wild populations.

## Conclusion

Very few studies have investigated variation in fungal GM structure across individuals in wild animal populations, despite the known impact of fungal species on host health in captivity. In this study, we identified substantial individual variation in the fungal GM of Seychelles warblers on Cousin Island. Understanding the relative importance of environmental factors and host genetics in driving such variation is crucial if we are to understand the dynamics of the GM and its potential influence on host evolution. We show that the fungal GM of the Seychelles warbler is primarily sculpted by environmental factors. Fungal communities were particularly distinct between the major and minor breeding seasons, although it is not yet clear whether variation in climatic variables (e.g. rainfall) or reproductive activity underlie these differences. In contrast, host genetic variation had a smaller impact on the fungal GM. The presence of certain MHC alleles was associated with differences in fungal GM alpha diversity, and there was some evidence that MHC-I diversity had an impact on fungal GM composition. However, further work is needed to determine the mechanisms underlying these associations. Fungal GM composition also differed between individuals that survived and those that died by the next breeding season suggesting some link between the fungal GM and host fitness. However, further work is needed to establish the causality of such relationships, or whether changes in the fungal GM are correlated with differences in host habitat and/or condition. Defining the function of specific fungal taxa in the GM and the importance of transient strains will also play a role in establishing this. Furthermore, a holistic approach that integrates information on other microbial kingdoms in the GM and quantifies covariation between the abundances of bacterial and fungal strains will be essential if we are to fully understand GM dynamics and the importance of different GM components to host fitness.

## Supplementary Information

**Additional file 1:** 

**Additional file 2:** 

## Data Availability

All ITS2 gene amplicon sequences have been submitted to the European Nucleotide Archive (ENA) database under the study accession number PRJEB54641. All 16S rRNA gene amplicon sequences have been submitted to the ENA database under the study accession numbers PRJEB45408 (samples taken in 2017 and 2018) and PRJEB47095 (samples taken in 2019). The GenBank accession number for MHC class I alleles is MZ509455-74 and for MHC class II alleles is MZ509475-98. The scripts and metadata to reproduce all analyses and figures can be accessed via the GitHub repository, https://github.com/Seychelle-Warbler-Project.
