# Supplementary material for: Assessing the causes and consequences of gut mycobiome variation in a wild population of the Seychelles warbler
Source: Microbiome. 2022 Dec 28;10:242. doi: 10.1186/s40168-022-01432-7 (PMC9795730; doi:10.1186/s40168-022-01432-7)
Supplement: Supplementary file 2 — Additional file 1: [file 40168_2022_1432_MOESM1_ESM.pdf]

Additional File 1

Table S1 Primers used to amplify the ITS2 subregion of the fungal internal transcribed spacer (ITS) ribosomal DNA.

Primer name	Sequence	Reference
gITS7_fw	5’- GTGARTCATCGARTCTTTG-3’	Ihrmark et al., 2012 [75]
ITS4_rev	5’- TCCTCCGCTTATTGATATGC-3’	White, Innis, Gelfand, & Sninsky, 1990 [76]

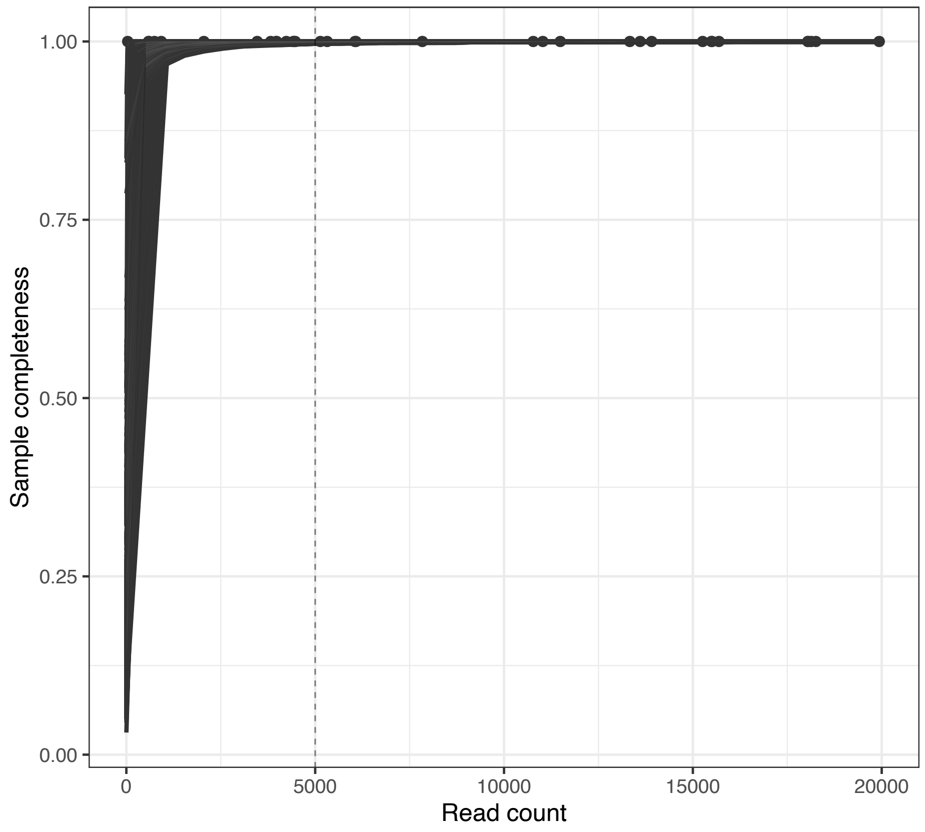

Fig. S1 Sample completeness curves in relation to number of reads. Curves were generated using the R package iNEXT 2.0.20, with 50 bootstrap replicates per sample. The vertical dashed line represents the number of reads used as a cut-off for retaining samples in downstream analysis (all samples with fewer than 5,000 reads were removed from the analysis).

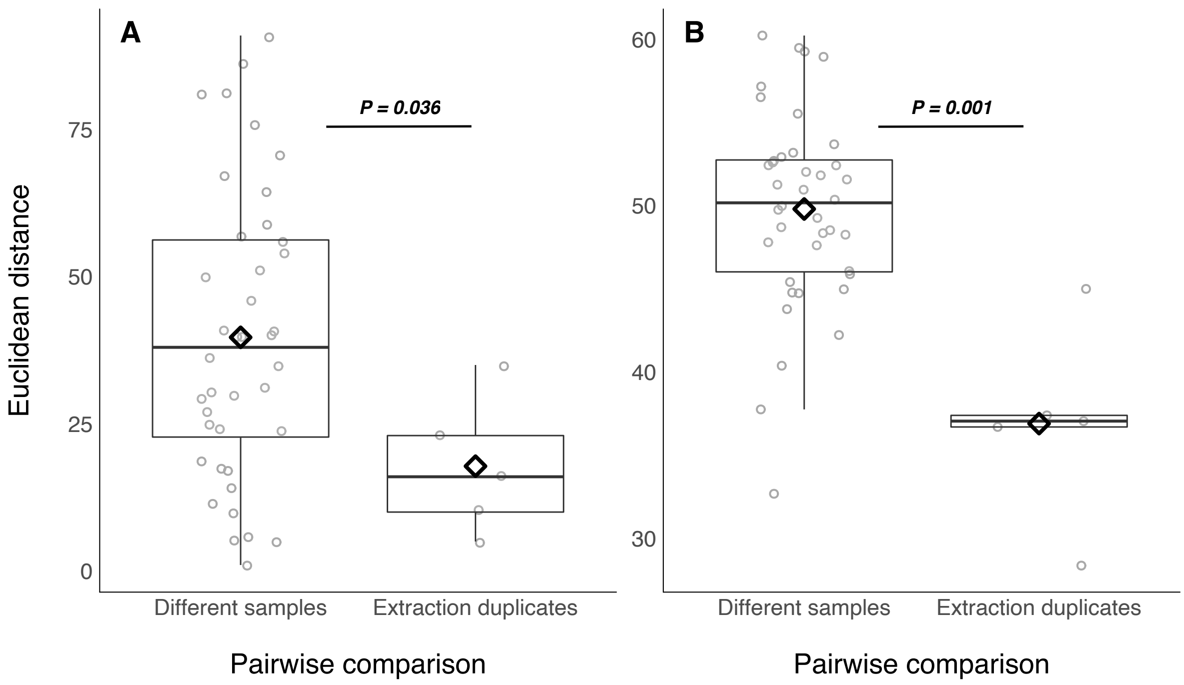

Fig. S2 The similarity of A) alpha, and B) beta diversity measures across DNA extractions of Seychelles warbler faecal samples. Pairwise Euclidean distances were calculated between different DNA extractions of the same faecal sample (n=5 extraction duplicates), and for all pairwise combinations of the five different faecal samples. Alpha diversity was calculated as observed ASV richness and beta diversity was calculated using CLR-transformed ASV abundances. Boxes span the interquartile (25% - 75%) range. The median is marked by a horizontal line and the mean is marked by a diamond. Whiskers extend to 1.5 times the interquartile range. Significant differences are shown; *P*-values were derived from a Kruskal-Wallis tests.

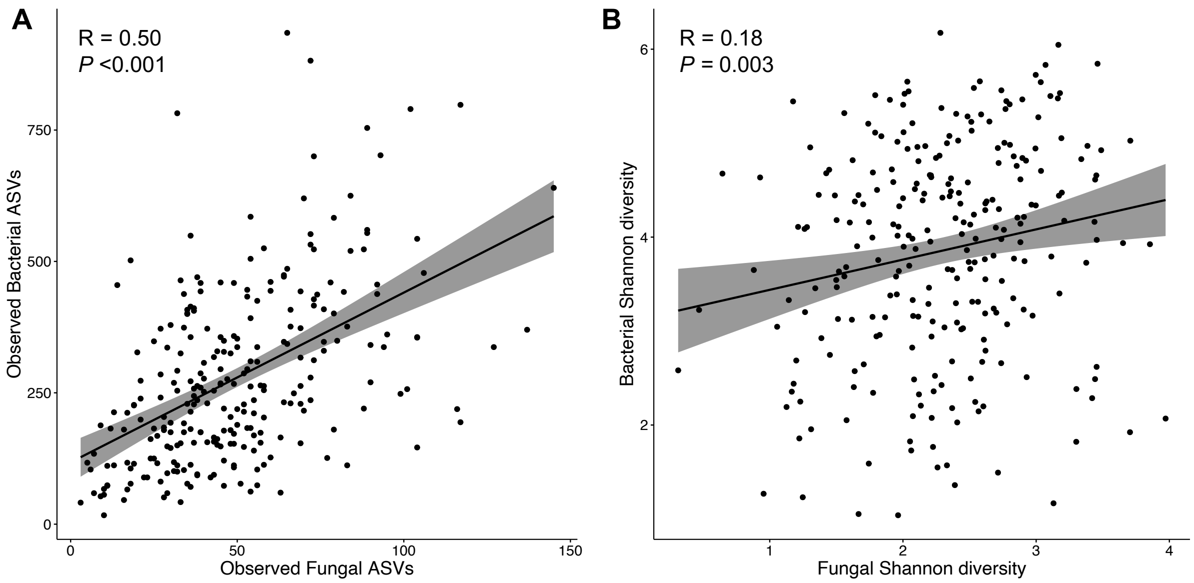

Fig. S3 Correlation between bacterial and fungal A) ASV richness and B) Shannon diversity in Seychelles warbler faecal samples (n = 257). The line of best fit and 95% confidence intervals are shown, as well as the results of a Pearson's correlation test.

Table S2 Core fungal A) Families, B) Genera, and C) Amplicon Sequencing Variants (ASVs) identified in Seychelles warbler faecal samples. Core taxa were identified as those that were present in at least 50% of samples with a minimum relative abundance of 0.1%. The mean relative abundance across all samples (n = 259) ± the standard deviation (SD) is shown for each taxon, as well its prevalence (the percentage of samples the taxon was detected in).

Taxon	Mean relative abundance (%) ± SD	Prevalence (%)
**A) Core Families**		
Cladosporiaceae	34.11 ± 23.29	98.07
Dothideales fam Incertae sedis	10.79 ± 11.96	93.82
Aspergillaceae	7.43 ± 12.72	90.35
Bionectriaceae	3.49 ± 8.56	87.64
Didymellaceae	3.17 ± 7.01	67.57
Microstromatales fam Incertae sedis	5.19 ± 7.12	67.18
Bulleribasidiaceae	1.02 ± 4.27	65.64
Nectriaceae	2.46 ± 5.93	64.86
Phaeosphaeriaceae	1.69 ± 4.44	64.09
Hypocreales fam Incertae sedis	2.12 ± 5.51	62.93
Unidentified (order *Capnodiales)*	1.84 ± 5.07	61.78
Unidentified (phylum *Ascomycota)*	0.92 ± 2.79	55.60
Neodevriesiaceae	1.41 ± 3.56	51.35
**B) Core Genera**		
Cladosporium	34.03 ± 23.27	98.07
Hortaea	10.79 ± 11.96	93.82
Penicillium	3.79 ± 8.78	90.35
Gliomastix	2.54 ± 7.45	72.97
*Unidentified (*family *Didymellaceae*)	3.04 ± 6.74	71.81
Sympodiomycopsis	4.92 ± 6.78	64.48
Aspergillus	3.56 ± 8.79	62.93
Vishniacozyma	0.92 ± 4.26	57.92
Fusarium	1.69 ± 5.38	57.14
Acremonium	1.42 ± 3.30	56.76
Unidentified (order *Capnodiales*)	1.84 ± 5.07	51.35
Unidentified (phylum *Ascomycota*)	0.92 ± 2.79	51.35
Neodevriesia	1.20 ± 3.48	50.58
**C) Core ASVs**		
Cladosporium dominicanum	28.57 ± 20.77	96.91

Hortaea werneckii	9.25 ± 10.85	91.51
Cladosporium coloradense	3.06 ± 5.90	76.83
Penicillium citrinum	2.14 ± 6.31	67.57
Unidentified *(family Didymellaceae)*	2.19 ± 5.38	63.32
Sympodiomycopsis kandeliae	1.96 ± 3.03	61.00
Sympodiomycopsis kandeliae	1.23 ± 2.55	58.69

Table S3 The impact of MHC diversity and TLR3 genotype on fungal alpha diversity in the gut microbiome of the Seychelles warbler. A) Shannon diversity and B) Observed ASV richness were used as the response variables in two separate models. Estimates and standard errors are based on conditional model-averaged estimates. Significant ($P < 0.05$) predictors are shown in bold. The reference categories for categorical variables were as follows: female (sex), major (season), AM (time of day), and $TLR3^{AA}$ (*TLR3* genotype). N= 189 samples/individuals were included in the analysis.

Predictor	Estimate	SE	*z*	*P*
**A) Shannon diversity**				
**(Intercept)**	**2.212**	**0.066**	**33.324**	**<0.001**
Age	-0.033	0.090	0.362	0.717
Sex (male)	0.052	0.089	0.580	0.562
Heterozygosity	-0.025	0.088	0.276	0.782
**Season (minor)**	**0.390**	**0.110**	**3.521**	**<0.001**
Territory quality	-0.044	0.089	0.493	0.622
Time of day (PM)	-0.103	0.091	1.126	0.260
Time stored at 4 °C	0.132	0.091	1.438	0.150
MHC-I diversity	-0.172	0.089	1.923	0.055
MHC-II diversity	0.095	0.093	1.009	0.313
TLR3 genotype				
$TLR3^{AC}$	-0.073	0.094	0.778	0.437
$TLR3^{CC}$	-0.096	0.173	0.553	0.580
**B) Observed ASV richness**				
**(Intercept)**	3.923	0.060	64.802	<0.001
Age	-0.082	0.076	1.064	0.287
Sex (male)	-0.040	0.076	0.521	0.602
Heterozygosity	-0.068	0.076	0.892	0.372
Season (minor)	0.152	0.093	1.618	0.106
Territory quality	-0.047	0.078	0.597	0.551
Time of day (PM)	-0.122	0.078	1.555	0.120
Time stored at 4 °C	-0.060	0.081	0.742	0.458
MHC-I diversity	-0.136	0.075	1.788	0.074

MHC-II diversity	0.032	0.080	0.392	0.695
TLR3 genotype				
$TLR3^{AC}$	-0.063	0.079	0.790	0.430
$TLR3^{CC}$	-0.126	0.148	0.846	0.397

Table S4 The effect of specific MHC alleles and *TLR3* genotype on fungal alpha diversity in the gut microbiome of the Seychelles warbler, controlling for other host and environmental variables. Diversity was measured as A) Shannon diversity and B) observed ASV richness. N= 189 individuals were included in the analysis. Estimates and standard errors are based on linear conditional model-averaged estimates. Significant predictors ($P < 0.05$) are highlighted in bold. The reference categories for categorical variables were as follows: female (sex), major (season), AM (time of day), $TLR3^{AA}$ (*TLR3* genotype), and absent (for all MHC alleles).

Predictor	Estimate	SE	*z*	*P*
**A) Shannon diversity**				
**(Intercept)**	**2.341**	**0.135**	**17.316**	**<0.001**
Age	-0.030	0.088	0.341	0.733
Sex (male)	0.040	0.088	0.451	0.652
Heterozygosity	-0.007	0.088	0.083	0.934
**Season (minor)**	**0.385**	**0.110**	**3.470**	**0.001**
Territory quality	-0.036	0.090	0.399	0.690
Time of day (PM)	-0.091	0.091	0.992	0.321
Time stored at 4 °C	0.136	0.091	1.484	0.138
TLR3 genotype				
$TLR3^{AC}$	-0.046	0.092	0.496	0.620
$TLR3^{CC}$	-0.073	0.174	0.416	0.678
Ase-ua1	-0.159	0.126	1.254	0.210
Ase-ua3	-0.050	0.158	0.314	0.753
Ase-ua4	-0.192	0.140	1.363	0.173
Ase-ua5	-0.080	0.148	0.541	0.588
Ase-ua6	0.000	0.115	0.003	0.997
Ase-ua7	-0.114	0.163	0.695	0.487
Ase-ua8	0.168	0.129	1.296	0.195
Ase-ua9	-0.011	0.168	0.064	0.949
Ase-ua11	**-0.249**	**0.125**	**1.982**	**0.048**
Ase-dab3	0.176	0.127	1.378	0.168
Ase-dab4	-0.024	0.126	0.190	0.849
Ase-dab5	0.031	0.116	0.266	0.791

**B) Observed ASV richness**				
**(Intercept)**	**4.097**	**0.142**	**28.662**	**<0.001**
Age	-0.048	0.074	0.639	0.523
Sex (male)	-0.055	0.074	0.735	0.462
Heterozygosity	-0.053	0.075	0.704	0.482
Season (minor)	0.122	0.094	1.293	0.196
Territory quality	-0.056	0.076	0.740	0.459
Time of day (PM)	-0.115	0.076	1.503	0.133
Time stored at 4°C	-0.075	0.077	0.969	0.332
TLR3 genotype				
$TLR3^{AC}$	-0.050	0.077	0.640	0.522
$TLR3^{CC}$	-0.168	0.147	1.140	0.254
Ase-ua1	-0.099	0.114	0.864	0.388
Ase-ua3	0.018	0.131	0.138	0.890
Ase-ua4	**-0.265**	**0.123**	**2.149**	**0.032**
Ase-ua5	0.051	0.099	0.509	0.611
Ase-ua6	0.001	0.104	0.013	0.989
Ase-ua7	**-0.283**	**0.126**	**2.227**	**0.026**
Ase-ua8	**0.230**	**0.106**	**2.155**	**0.031**
Ase-ua9	0.103	0.125	0.820	0.412
Ase-ua11	-0.172	0.164	1.041	0.298
Ase-dab3	**0.232**	**0.108**	**2.144**	**0.032**
Ase-dab4	-0.154	0.106	1.439	0.150
Ase-dab5	-0.159	0.128	1.239	0.215

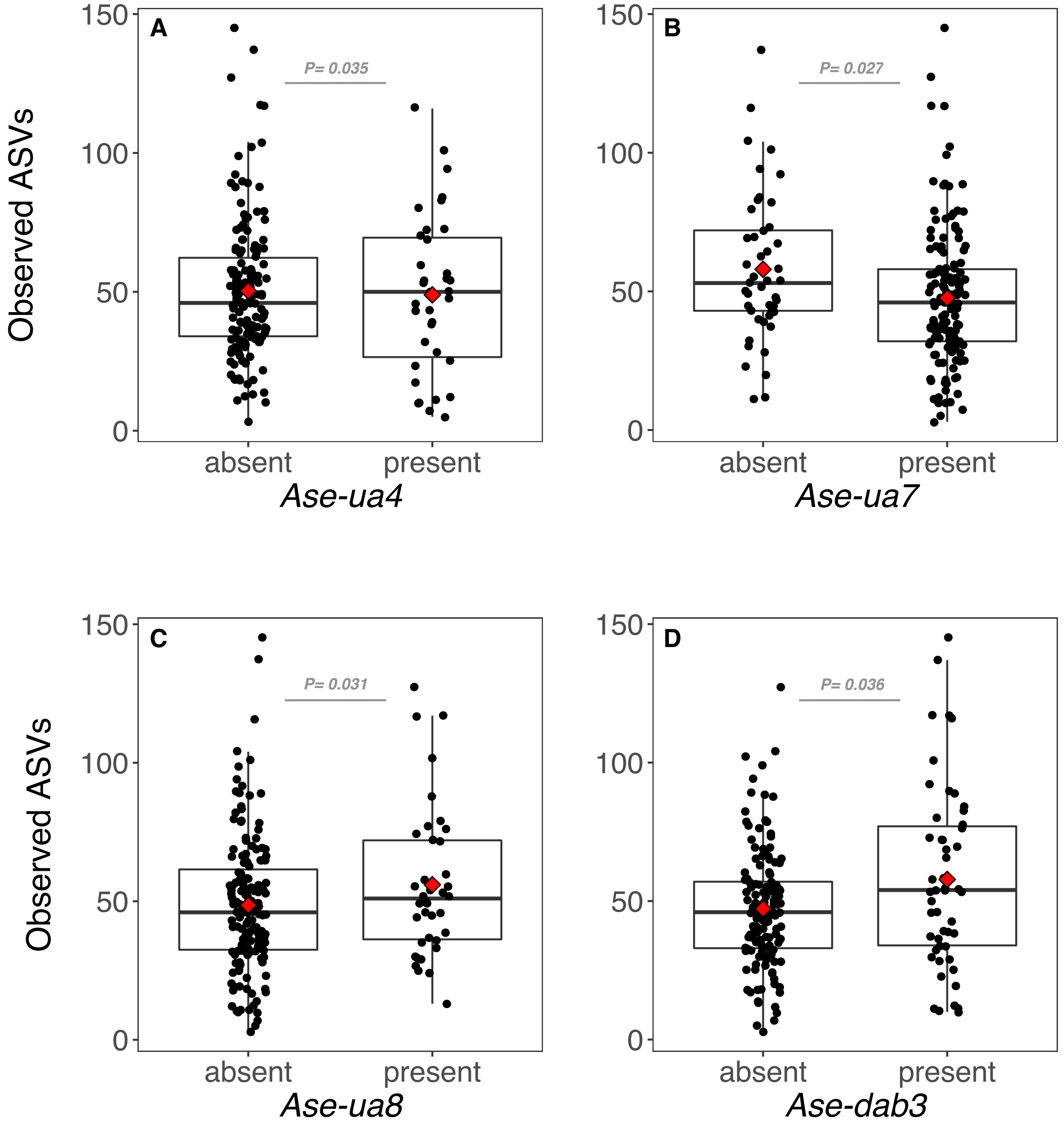

Fig. S4 The relationship between the presence/absence of MHC alleles and observed fungal ASV richness in the Seychelles warbler gut microbiome. Three MHC-I alleles (**A.** *Ase-ua4*, **B.** *Ase-ua7*, and **C.** *Ase-ua8*) and one MHC-II allele (**D.** *Ase-dab3*) were significantly associated with changes in observed ASV richness. Black points represent raw values. Boxes span the interquartile (25% - 75%) range. The median is marked by a horizontal line and the mean is marked by a red diamond. Whiskers extend to 1.5 times the interquartile range. *P*-values are based on linear conditional model-averaged estimates (see Table S5 for model results in full). N= 189 individuals were included in the analysis.

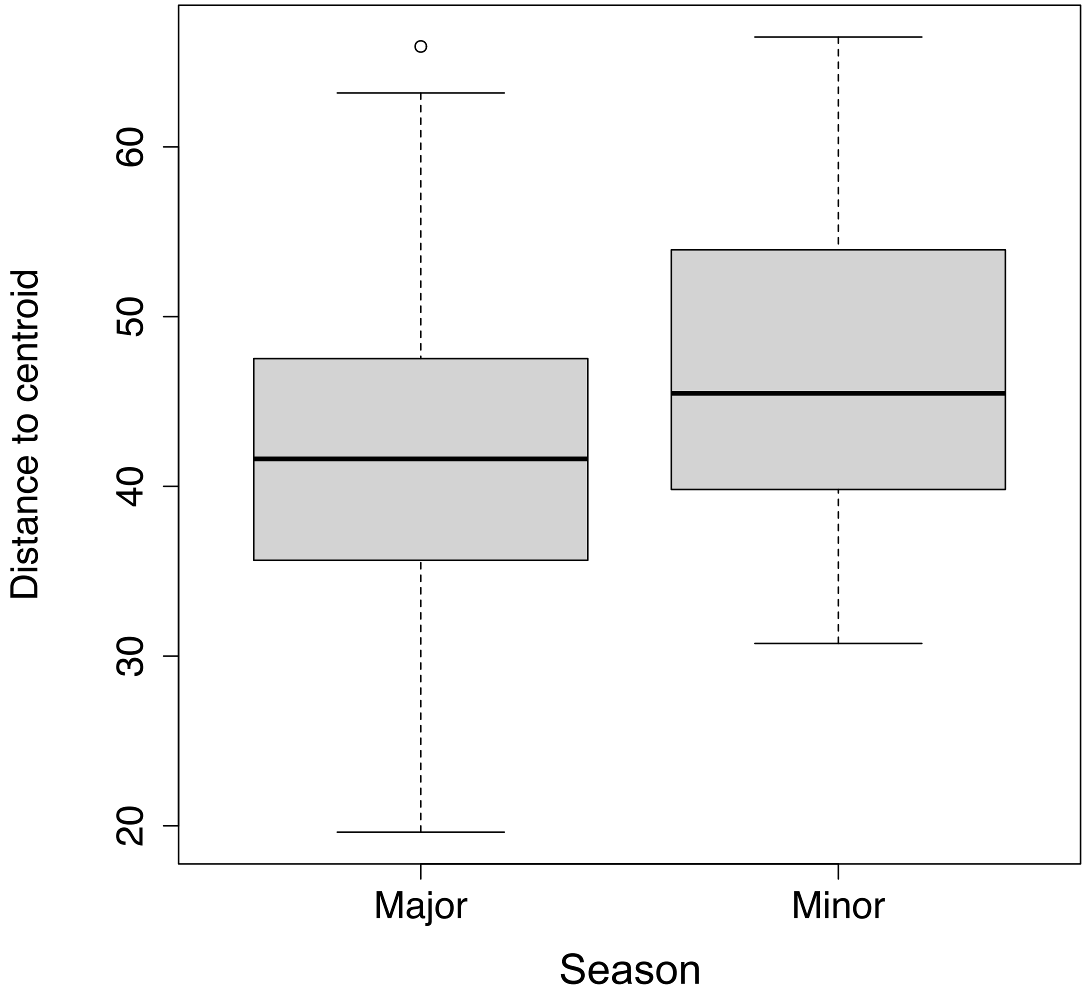

Fig. S5 Results of a *betadisper* analysis showing a significant difference ($F_{1,253} = 19.654$, $P < 0.001$) in fungal gut microbiome variability (distance to centroid) between sampling seasons in the Seychelles warbler. Boxes encompass the interquartile (25%-75%) range and the median is marked by a horizontal line. Whiskers extend to 1.5 times the interquartile range. Major season = 182 samples, minor season = 73 samples.

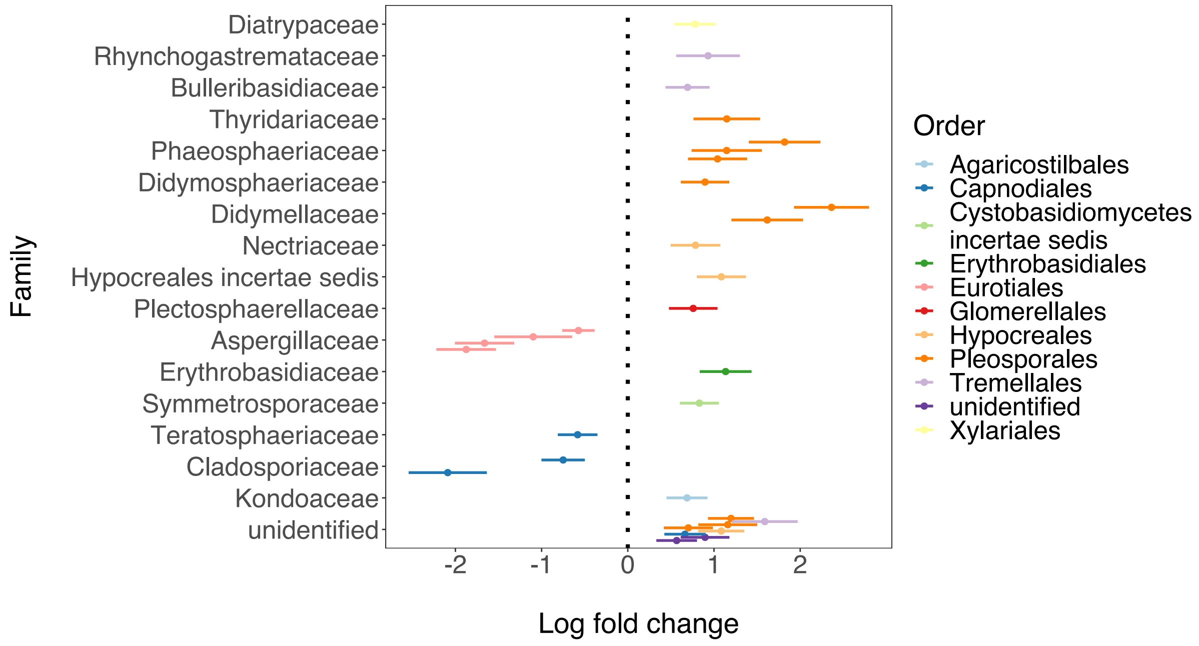

Fig. S6 Differentially abundant Amplicon Sequencing Variants (ASVs) in the gut microbiome of Seychelles warblers sampled in the minor or major breeding seasons, respectively. Points represent the log fold change (effect size) of individual fungal ASVs; only ASVs with significant effect sizes ($P_{adj} < 0.05$) are shown. A positive log fold change indicates that an ASV is significantly more abundant in individuals sampled in the minor breeding season, and a negative log fold change indicates a higher abundance in individuals sampled in the major breeding season. N = 255 samples were included in the analysis. Bars represent 95% confidence intervals derived from the ANCOM-BC model. ASVs are classified by fungal family on the y-axis and are coloured by fungal order. A full taxonomic breakdown and model outputs are presented in Additional file 2.

Table S5 The influence of specific MHC alleles on fungal gut microbiome beta diversity. The results are based on a PERMANOVA analysis of Euclidean distances, calculated using CLR transformed Amplicon Sequencing Variant (ASV) abundances. Significant predictors ($P < 0.05$) are shown in bold. The analysis included 189 samples/individuals.

**Predictor**	***df***	R^2	***F***	***P***
Age	1	0.005	1.001	0.452
Sex	1	0.005	0.931	0.721
Heterozygosity	1	0.005	0.970	0.574
**Season**	**1**	**0.011**	**2.029**	**<0.001**
**Territory quality**	**1**	**0.008**	**1.473**	**0.003**
Time of day	1	0.005	1.040	0.324
**Time stored at 4 °C**	**1**	**0.009**	**1.648**	**<0.001**
**Survival**	**1**	**0.008**	**1.452**	**0.005**
TLR3 genotype	2	0.010	0.980	0.559
Ase-ua1	1	0.005	0.888	0.830
Ase-ua3	1	0.005	0.990	0.499
Ase-ua4	1	0.006	1.065	0.260
Ase-ua5	1	0.005	0.952	0.625
Ase-ua6	1	0.005	1.006	0.443
Ase-ua7	1	0.005	1.017	0.406
Ase-ua8	1	0.005	0.967	0.584
Ase-ua9	1	0.006	1.110	0.173
Ase-ua11	1	0.006	1.157	0.099
Ase-dab3	1	0.005	0.919	0.741
Ase-dab4	1	0.005	0.907	0.786
Ase-dab5	1	0.004	0.833	0.941

Table S6. The association between gut microbiome alpha diversity and survival to the next breeding season in the Seychelles warbler. A separate model was run for each metric of alpha diversity; **A)** Shannon diversity and **B)** observed ASV richness. N = 255 samples/individuals were included in the analysis (218 individuals survived, 37 individuals died). Estimates and standard errors are based on linear conditional model-averaged estimates. Significant predictors (*P* < 0.05) are highlighted in bold. The reference categories for categorical variables were as follows: female (sex), major (season), 2017 (sample year).

**Predictor**	**Estimate**	**SE**	***z***	***P***
**A) Shannon diversity**				
**(Intercept)**	**1.967**	**0.377**	**5.198**	**<0.001**
Age	0.631	0.455	1.380	0.168
Sex	-0.534	0.369	1.438	0.150
Shannon diversity	0.427	0.362	1.174	0.240
Territory quality	0.895	0.537	1.657	0.098
Season	0.009	0.437	0.020	0.984
Sample Year				
2018	0.268	0.764	0.349	0.727
2019	0.045	0.787	0.057	0.954
**B) Observed ASV richness**				
**(Intercept)**	**1.956**	**0.373**	**5.229**	**<0.001**
Age	0.615	0.452	1.352	0.176
Sex	-0.530	0.368	1.431	0.152
ASV richness	0.012	0.357	0.032	0.974
Territory quality	0.872	0.533	1.628	0.104
Season	0.074	0.423	0.175	0.861
Sample Year				
2018	0.225	0.762	0.294	0.769
2019	0.018	0.786	0.023	0.981
